# Ecdysteroid responses to urban heat island conditions during development of the western black widow spider (*Latrodectus hesperus*)

**DOI:** 10.1371/journal.pone.0267398

**Published:** 2022-04-28

**Authors:** Claire Moen, J. Chadwick Johnson, Jennifer Hackney Price

**Affiliations:** School of Math & Natural Sciences, Arizona State University—West Campus, Glendale, AZ, United States of America; Northeastern University (Shenyang China), CHINA

## Abstract

The steroid hormone 20-hydroxyecdysone (20E) controls molting in arthropods. The timing of 20E production, and subsequent developmental transitions, is influenced by a variety of environmental factors including nutrition, photoperiod, and temperature, which is particularly relevant in the face of climate change. Environmental changes, combined with rapid urbanization, and the increasing prevalence of urban heat islands (UHI) have contributed to an overall decrease in biodiversity making it critical to understand how organisms respond to elevating global temperatures. Some arthropods, such as the Western black widow spider, *Latrodectus hesperus*, appear to thrive under UHI conditions, but the physiological mechanism underlying their success has not been explored. Here we examine the relationship between hemolymph 20E titers and spiderling development under non-urban desert (27°C), intermediate (30°C), and urban (33°C) temperatures. We found that a presumptive molt-inducing 20E peak observed in spiders at non-urban desert temperatures was reduced and delayed at higher temperatures. Intermolt 20E titers were also significantly altered in spiders reared under UHI temperatures. Despite the apparent success of black widows in urban environments, we noted that, coincident with the effects on 20E, there were numerous negative effects of elevated temperatures on spiderling development. The differential effects of temperature on pre-molt and intermolt 20E titers suggest distinct hormonal mechanisms underlying the physiological, developmental, and behavioral response to heat, allowing spiders to better cope with urban environments.

## Introduction

Extreme temperature fluctuations associated with global climate change are exposing organisms to an unprecedented level of temperature stress [1–3]. Temperatures exceeding optimum can have multiple negative consequences including, but not limited to, immune system dysfunction [4], increased mortality rates [5–8], decreased fertility [9, 10], developmental delays [7, 8], and slower growth rates [10–12]. Compounding the effects of climate change is the phenomenon of urbanization [10]. Approximately 55% of humans live in cities and that number is expected to increase to 68% by 2050 [13]. Urban development can affect organisms in several different ways. Urbanization causes habitat fragmentation or loss and the introduction of nonnative species [14]. In addition, the Urban Heat Island (UHI) effect, which is caused by built structures (e.g. concrete and asphalt) within urban cities capturing heat during the day and retaining it throughout the night, results in warmer temperatures in developed areas in comparison to the surrounding rural environment [15, 16]. While the intensity of the thermal environment varies from city to city and between seasons and can be influenced by numerous factors such as 3D architecture and urban ventilation, elevated UHI temperatures and other factors associated with urbanization negatively affect many animals, contributing to an overall decrease in biodiversity in urban areas when compared to surrounding non-urban areas [17–26].

Some organisms, termed urban exploiters, thrive in urban environments, where they are often found at high densities in developed areas compared to surrounding nonurban regions [27]. Herring gulls (*Larus argentatus*), blackbirds (*Turdus merula*), house mice (*Mus musculus*), brown rats (*Rattus norvegicus*), and feral pigeons (*Columba livia*) are just some examples of vertebrate urban exploiters [28–34]. The effects of urbanization observed in vertebrates is mirrored in invertebrates, with urban populations being less diverse than those in rural environments while urban exploiters thrive in the face of urban change [35, 36]. For example, under urban conditions (increased temperature and decreased humidity), the wall brown butterfly (*Lasiommata megera*) demonstrates increased larval survival and the production of larger adults [37]. Similarly, urban humped golden orb-weaving spiders (*Nephila plumpipes*) are larger and produce more offspring than their rural counterparts [38].

One urban exploiter, the western black widow spider (*Latrodectus hesperus*), is flourishing in the face of urbanization [39, 40]. Black widows are found throughout the desert southwest including the Sonoran Desert and thrive in urban areas even though urban centers are significantly warmer than the surrounding non-urban desert [40]. Previous research has shown in the summer, during which females can produce several egg sacs, the average temperatures for arthropod microclimates in urban Phoenix, Arizona to be elevated by 6°C compared to average desert temperatures (urban = 33°C vs desert = 27°C; [7]). Even with these elevated urban temperatures, *L*. *hesperus* is found at densities that are 30 times more concentrated than their rural counterparts [40]. Despite this apparent success, we have recently shown that urban temperatures are detrimental to spider development [7]. Elevated temperatures increase spider mortality, increase the time between molts, and decrease growth rates leading to the production of smaller adults [7]. Spiders alter certain behaviors (e.g. decreased web building and increased aggression) under urban conditions, which, along with increased food availability, are thought to partially offset the negative effects of UHI temperatures [7, 40, 41]. However, understanding the physiological responses to urbanization may help us to better understand the success of these arachnids in urban environments in the face of the negative consequences of urban temperatures. Understanding the impact of rising temperatures on animal physiology will be of extreme importance as global temperatures continue to rise, with urban heat models being an early predictor of viability of organisms in the rapidly changing environment.

Molting in arthropods is regulated primarily by the steroid hormone 20-hydroxyecdysone (20E), which initiates gene expression cascades leading to the physiological, morphological, and behavioral changes associated with major developmental transitions such as molting, during which the larval cuticle is shed and replaced with a new, larger cuticle allowing for organism growth [42–49]. Ecdysteroid regulation of molting and metamorphosis has been extensively studied in a wide variety of insects including *Drosophila melanogaster*, *Bombyx mori*, and *Manduca sexta* where a steep rise in the hemolymph ecdysteroid titer precedes each major developmental transition [49–56]. In contrast, while ecdysteroids have been identified in arachnids, relatively little research has been done to document ecdysteroid titers during spider development [57–61].

Modulation of hormone titers and signaling cascades are thought to modify the timing of development (e.g. time between transitions such as molts and metamorphosis) in response to environmental changes including fluctuations in temperature, photoperiod, population density, and nutrition [62–73]. For example, in the desert toad (*Scaphiopus couchii*), habitat desiccation is associated accelerated metamorphosis due to an increase in cortisol and thyroid hormone titers [74]. In the fruit fly (*Drosophila melanogaster*), nutritional shortages and elevated temperatures each lead to developmental arrests in egg development associated with increased circulating ecdysteroid titers [75–79]. Ectopic ecdysteroid administration in *Drosophila* suppresses egg development and egg laying [80, 81]. Together, these studies suggest that in adult flies, stress-induced ecdysteroid production may lead to physiological changes that shifts energy allocation from reproduction to survival [75–80]. Elevated ecdysteroid titers induced by thermal stress also regulates a subset of small heat shock proteins such as Hsp23 which is thought to play a neuroprotective role in response to stress [82].

Here we examine how temperatures associated with Urban Heat Islands influence ecdysteroid titers during early spider development. We reared spiders under three different temperatures that mimic non-urban desert, urban, and intermediate environments. Because UHI temperatures are associated with developmental delays, we hypothesized that heat would likely delay the surge of ecdysteroids that precedes and stimulates molting, hereafter referred to as the ‘molting peak’. Because thermal stress is also associated with an overall increase in ecdysteroids in arthropods, we also hypothesized that high temperatures would result in basal ecdysteroid titers, which we refer to as the ‘intermolt ecdysteroid titers’, being increased throughout spider development. Our results confirm that UHI temperatures have negative effects on development (e.g. size, growth rate, mortality, time between molts) and are associated with changes in the ecdysteroid titers throughout spiderling development.

## Materials and methods

### Ethics statement

This study did not utilize animals requiring approval by an institutional review board or ethics committee. No field permits were involved. No other permissions were necessary for the research reported here.

### Sample collection

Adult female *L*. *hesperus* were collected from six collection sites across the urban Phoenix area (S1 Fig). Spiders were housed individually at 24°C and fed one cricket on a weekly basis. For ten females, 100–250 eggs from their first egg sac were individually reared in 4.13 x 4.13 x 5.56 cm enclosures containing two toothpicks measuring 6.3 cm crossing diagonally to provide a structure for web building. For the first 30 days of development, eggs were reared at room temperature (24°C) at a 12:12 photoperiod.

### Spider rearing

Starting 30 days after egg sac production each spider was fed two *Drosophila melanogaster* twice a week. Each spider was checked daily for molting and survival, which were recorded to track developmental progression, with shed cuticles being removed from enclosures when found. On day 44 of development, which includes 30 days of incubation in the egg sac and 14 days post-hatch, families were divided into environmental chambers (Percival Scientific), simulating temperature conditions of interest, which included 27, 30 and 33°C. Temperatures were chosen based on previous studies that demonstrated that in the summer, the average temperatures for arthropod microclimates in urban Phoenix, Arizona is elevated by 6°C compared to average desert temperatures (urban = 33°C vs desert = 27°C; [7]). Before day 44 of development, spiderlings transferred to higher temperatures after being removed from the egg sac do not typically survive (C. Moen, unpublished observation). In addition, inconsistencies were observed in the timing of molts prior to day 44 of development, when competition is highest between peers. Developmental data [molts, deaths, and mass] were recorded daily until day 75 of development. Data was not collected after day 75 of development as differences between males and females become noticeably different after day 75 (C. Moen, unpublished observation). Due to the addition of a 3^rd^, intermediate temperature (30˚C) during the second year of this study, only 6 of the 10 spider families were subjected to this temperature.

### Hormone extraction

Spiders were individually weighed and preserved in 200 μL of methanol. Samples were homogenized using plastic pestles and centrifuged for 20 minutes at 18,000 x g. Supernatants were collected while remaining insoluble material was again homogenized and centrifuged. The resulting supernatants were combined and dried using an Eppendorf Vacufuge. Dried samples (hormone extracts) were resuspended in 200 μL of EIA Buffer (0.4M NaCl, 1 mM EDTA, 0.1% BSA in 0.1M phosphate buffer), enough for two replicates to be carried out per spider.

### Measurement of hormone concentrations

Ecdysteroid concentrations were quantified using a competitive EIA (enzyme immunoassay) kit (Cayman Chemicals, Inc., USA) according to manufacturer’s instructions. In this assay, 20-hydroxyecdysone (20E) and 20E acetylcholinesterase (AChe), which were used as the standard and tracer respectively, compete for a limited number of binding sites on a rabbit anti-20E antibody. Ellman’s Reagent was used as the substrate, which was converted to a yellow product by AChe. Therefore, the production of product is inversely proportional to the amount of 20E in the sample. Standard curves were prepared with the 20E EIA Ecdysteroid Standard (Cayman Chemicals, Inc. USA) using concentrations ranging from 10 to 0.020 ng/μl. Because the antibody detects 20-hydroxyecdysone and its’ metabolites, we report 20E concentrations as 20E equivalents per mg tissue. The absorbance of samples and standards was measured at 415 nm using an ELX801U Ultra Microplate Reader (Bio-Tek Instruments). Microsoft Excel was used to analyze all resulting data. Absorbance data were linearized using a Logit transformation according to the following formula: (B/B_0_) = ln[B/B_0_/(1-B/ B_0_), where B represents the absorbance of samples or standards and B_0_ represents maximum binding, the absorbance obtained in samples containing all assay components except 20E. The standard curve was generated by plotting the logit-transformed data vs. log concentrations and linear regression analysis was used to determine the concentration of each spider sample. Resulting ecdysteroid concentrations were converted to pg 20E Equivalents/mg tissue to account for the mass of each spider. Ecdysteroid concentrations from 9 spiderlings did not fall within the limits of the 20E standard curve were not included in any subsequent analyses.

### Statistics

Data were analyzed via a One-Way ANOVA performed using the ANOVA Shiny web application available at istats.shinyapps.io/ANOVA. Shiny apps are interactive online data analysis and visualization tools that are created using Rstudio’s Shiny framework [83]. Pairwise comparisons were calculated using the Tukey multiple comparison test. Results were considered statistically significant if p ≤ 0.05 and marginally significant if p < 0.1. Figures were assembled in PowerPoint (Microsoft) and the GNU Image Manipulation Program (GIMP v2.10.10). Unless otherwise indicated, statistical data are presented as mean ± SEM.

## Results

### Assessment of ecdysteroids during development at different temperatures

**Fig 1:**
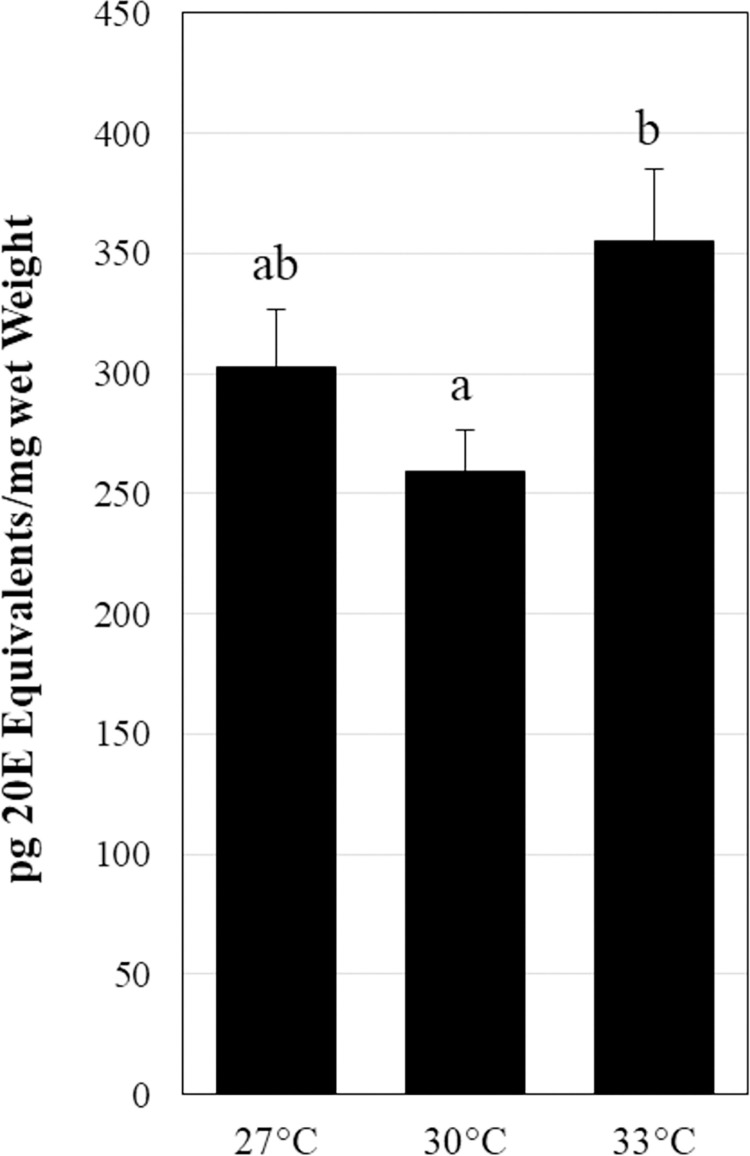
Average ecdysteroid titers during spiderling development. Ecdysteroid titers were determined for spiderlings reared at 27, 30, or 33˚C from 4 days before to 10 days after the second molt that occurred per family at 27˚C. Error bars represent standard error.

There was a significant effect of temperature on average ecdysteroid titers during development of Western black widow (*L*. *hesperus*) spiderlings (27˚C: desert = 302 ± 24 vs 30˚C: intermediate = 259 ± 17 vs 33˚C: urban = 355 ± 29 pg 20E/mg; F_2,211_ = 3.963, p = 0.0204; See Fig 1). Ecdysteroid titers at intermediate temperatures were similar to those measured at desert temperatures. However, urban temperatures were associated with significantly higher 20E titers than intermediate temperatures (T_140_ = -2.82, p = 0.01). While we detected a significant effect of spider family on average 20E titers at desert temperatures (F_9,103_ = 4.13, p = 0.0001; S2A Fig), ecdysteroid titers in all families appear to respond in a similar fashion to elevated temperatures. With few exceptions, relative to desert temperatures (27˚C), average ecdysteroid titers in each family were decreased at intermediate temperatures (30˚C) and increased at urban temperatures (33˚C) (S2B Fig).

**Fig 2:**
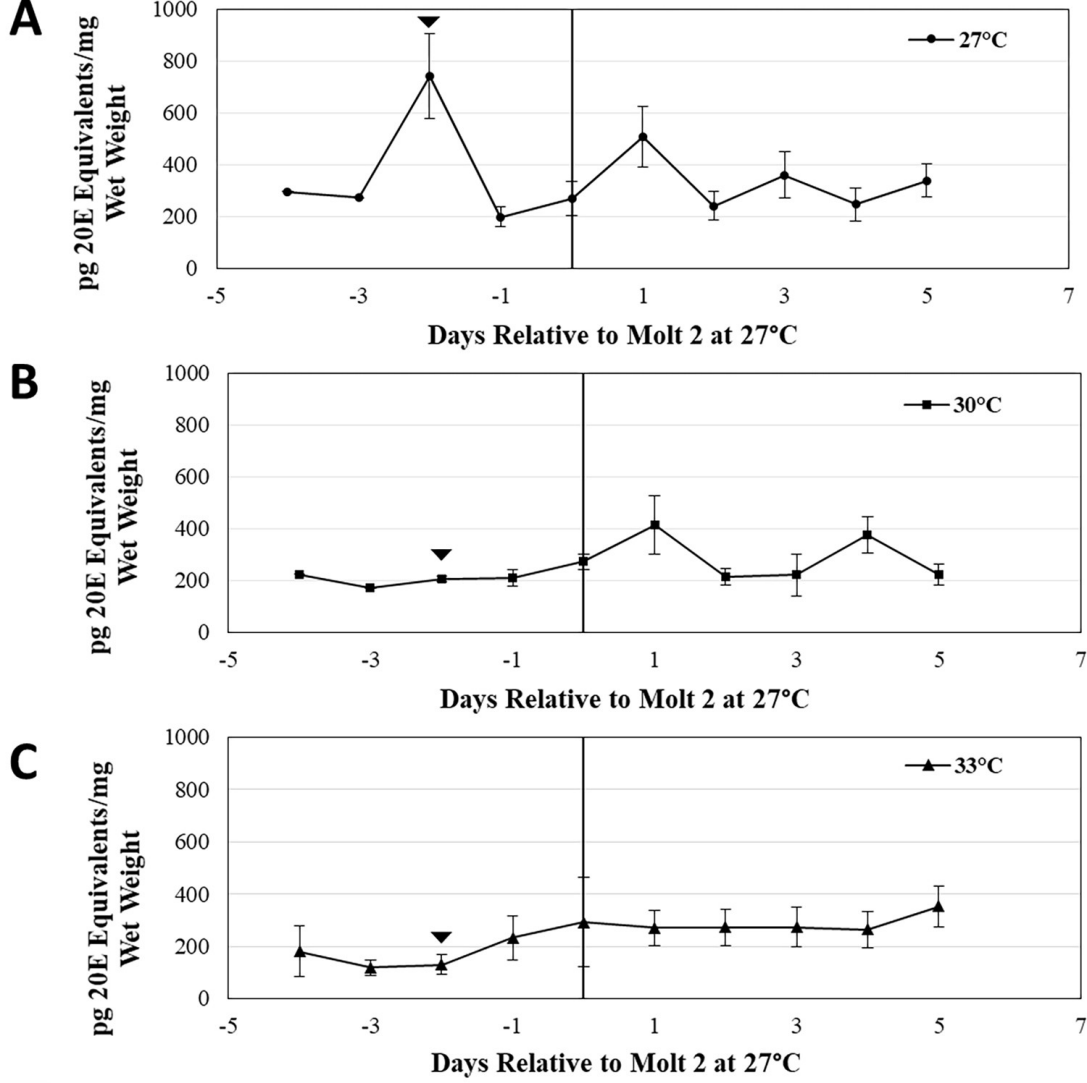
Developmental ecdysteroid profiles at different temperatures. (A-C) Average daily ecdysteroid titers from spiderlings reared at 27˚C (A), 30˚C (B), and 33˚C (C). Only families that had spiderlings reared at both temperatures were used for analysis. Spiderling ages are relative to the timing of the second molt for each family, at 27˚C. Error bars represent standard error.

Analysis of daily changes in ecdysteroid titers revealed that at 27°C, a sharp increase in ecdysteroid titers occurred two days prior to the second molt (Fig 2A). This large ecdysteroid peak was absent in spiderlings reared at 30°C (Fig 2B) and 33°C (Fig 2C). One day after the second molt, spiderlings from 27°C and 30°C treatments both exhibited a small pulse of ecdysteroids (Fig 2A and 2B). Additional small ecdysteroid peaks were visible in spiderlings at 30°C following the second molt, but these did not occur at the same time as those observed in spiderlings from the 27°C desert temperatures (Fig 2A).

**Fig 3:**
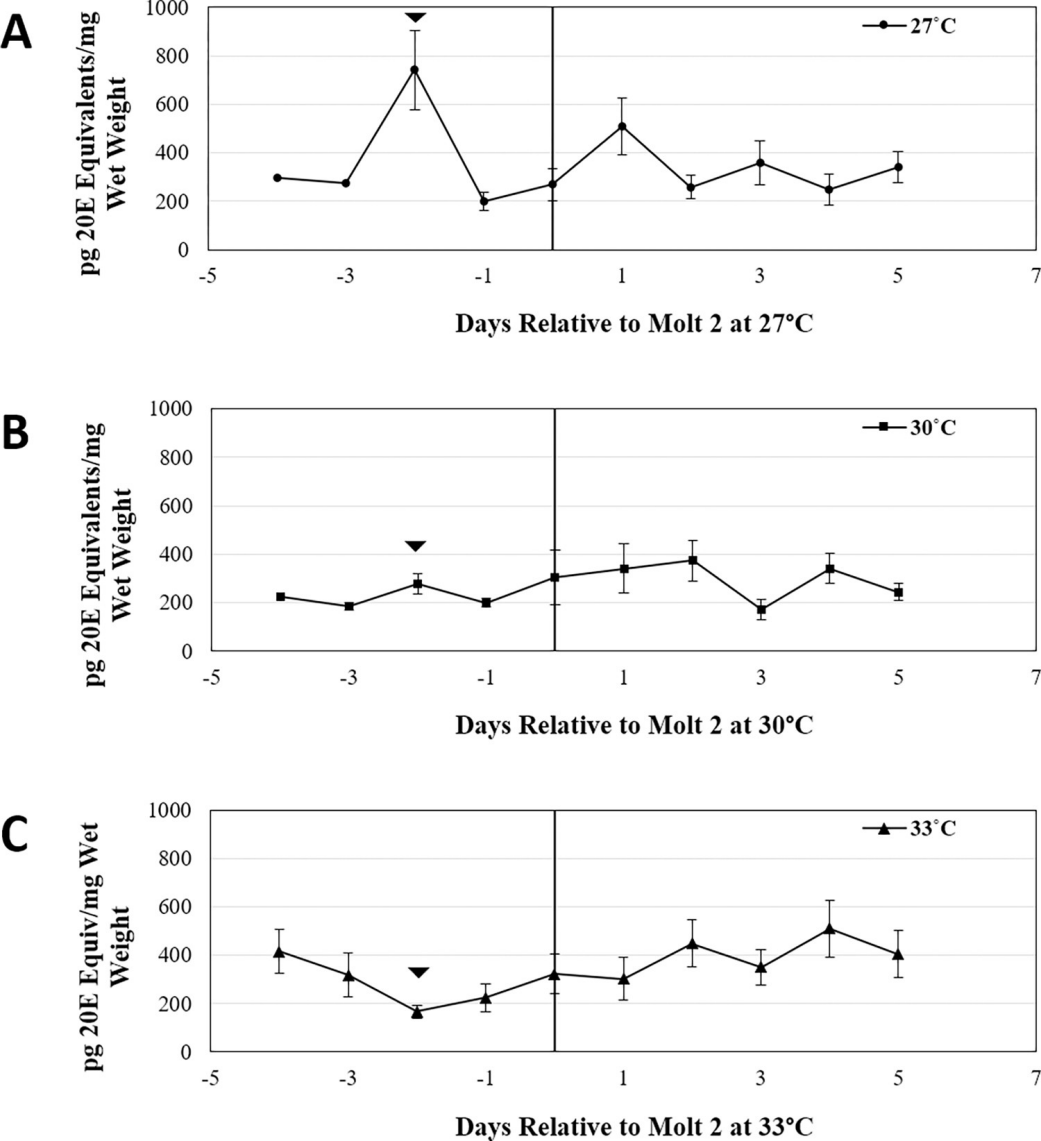
Normalized developmental ecdysteroid profiles. Average daily ecdysteroid titers from spiderlings reared at (A) 27˚C, (B) 30˚C and (C) 33˚C. Only families that had spiderlings reared at all three temperatures were used (N = 6 families). Spiderling ages are relative to the timing of the second molt for each family, at each temperature. The presumptive molt-inducing peak of ecdysone is indicated (arrowhead). Error bars represent standard error.

Because increased temperatures are known to alter the timing of the second molt [7], it is possible that pre-molt ecdysteroid peaks were obscured when spiderlings reared at 30°C and 33°C were normalized to molts occurring at 27°C. We therefore examined ecdysteroid developmental profiles in spiderlings staged based on the time at which the second molt occurred for each family at each temperature (Fig 3). Like what was observed at 27°C, this method revealed a small ecdysteroid peak occurring two days prior to the second molt in spiderlings reared at 30°C (Arrowhead; Fig 3A and 3B). In contrast, the pre-molt peak was not detected in spiderlings reared at 33°C (Arrowhead; Fig 3C).

**Fig 4:**
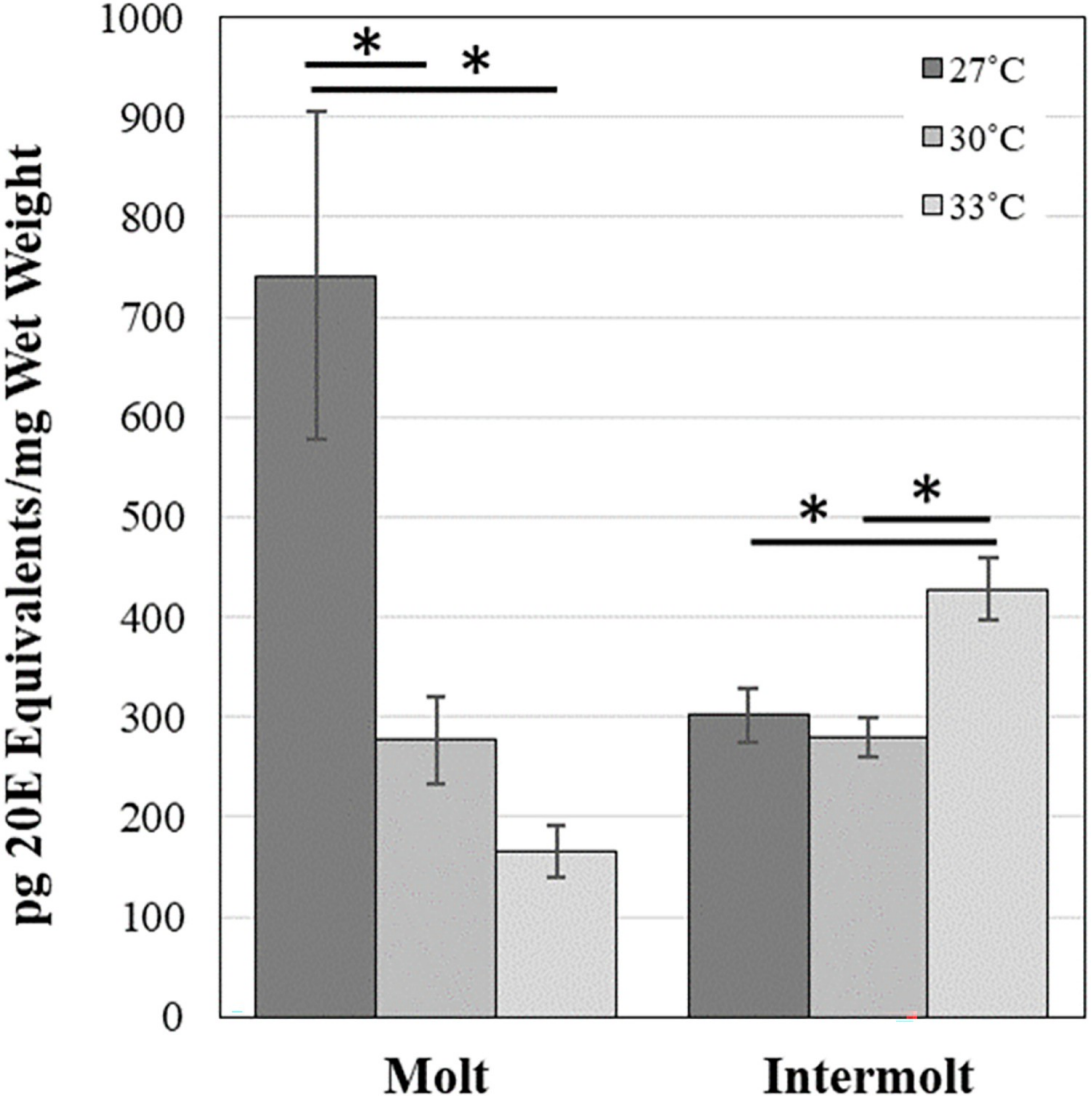
Phase-specific ecdysteroid titers. Average daily ecdysteroid titers from spiderlings reared at 27˚C, 30˚C and 33˚C were determined at two time points: two days prior to the second molt (Molt) and days 2–5 after the second molt (Intermolt). Only families that had spiderlings reared at all three temperatures were used (N = 6 families). Spiderlings were aged relative to the timing of the second molt for each family, at each temperature. Error bars represent standard error. (*) indicates a significant difference (p<0.05).

Analysis of ecdysteroid titers measured during two time intervals, one during the peak observed two days before the second molt (Fig 4, Molt) and the second spanning 2–5 days following the second mole (Fig 4, Intermolt), revealed a significant interaction between temperature and developmental phase (F_2,1_ = 10.723, p = 0.025). The molting peak at 27°C was significantly higher than that observed at both 30°C (T_4_ = 5.52, p = 0.000). and 33°C (T_5_ = 6.85, p = 0.000). No significant difference was observed between the molting ecdysteroid peak at 30°C and 33°C. In contrast, basal titers were significantly higher at 33°C when compared to 27°C (T_53_ = -2.68, p = 0.02) and 30°C (T_53_ = -3.18, p = 0.01).

### Assessment of developmental changes in response to various temperatures

**Fig 5:**
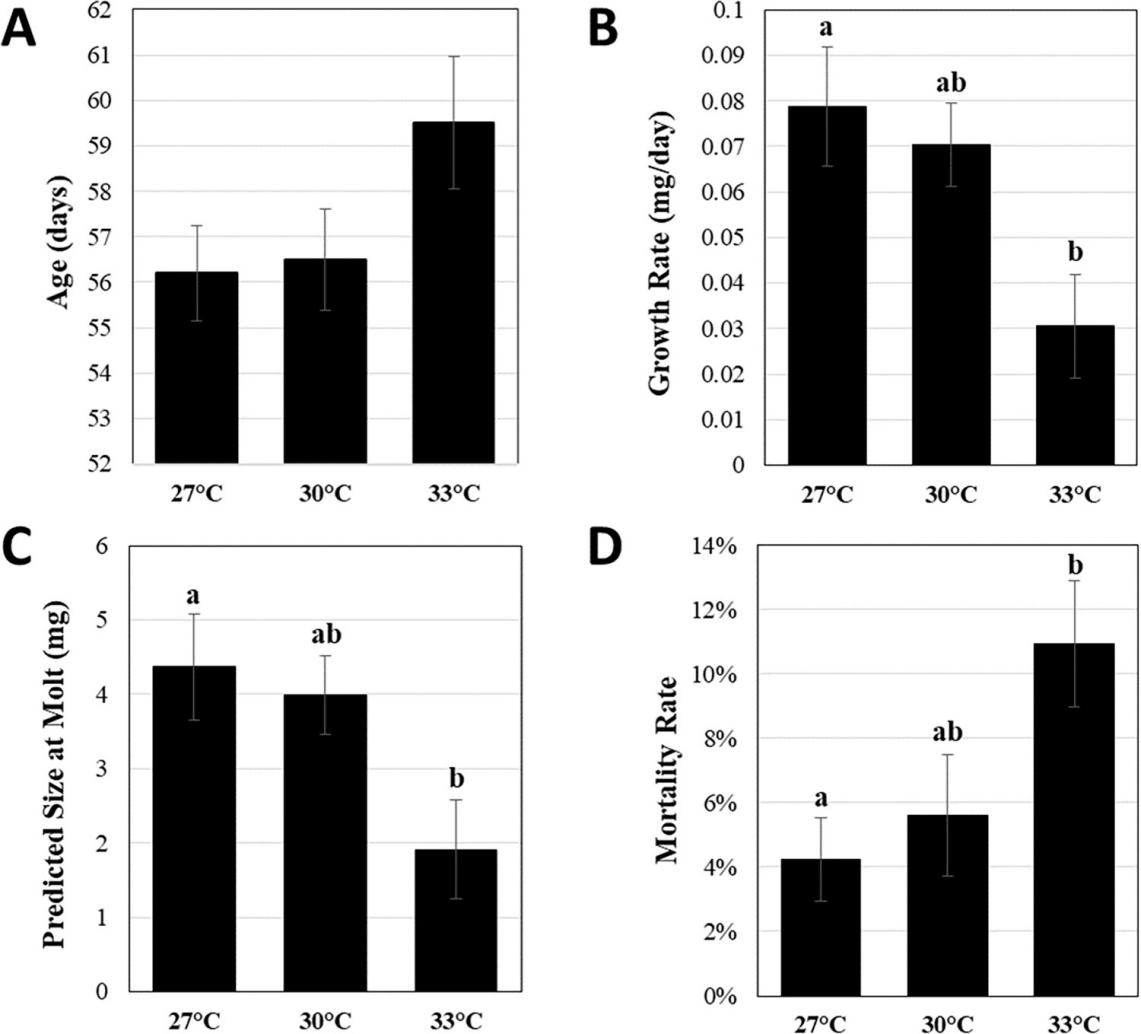
Effects of elevated temperatures on development. (A) Average molt times for spiderlings reared at different temperatures. (B) Average growth rates were measured from 55–75 days of development for spiderlings reared at different temperatures. (N = 6 families per treatment) (C) Average spiderling size at time of second molt was predicted from molt times and growth rates for each family (N = 6 families per treatment). (D) Spiderling mortality at each temperature was determined from 55–75 days of development for each family. (A-D) Different lowercase letters indicate significant differences (p<0.05). N = 6 families per treatment.

To better understand how different temperatures influence development, which is regulated by ecdysteroids in arthropods and arachnids, we analyzed several developmental metrics: timing of the second molt, growth rate, predicted size at the second molt, and mortality (Fig 5). Consistent with previous reports [7], we determined that spiderlings reared at 33°C underwent the second molt approximately three days later than siblings reared at 27°C and at 30°C (Fig 5A). However, the effects of temperature on the timing of the second molt were not statistically significant.

There was a significant effect of temperature treatments on spiderling growth rate (F_2,15_ = 5.191, p = 0.0194; Fig 5B). Spiders at 33°C grew significantly slower than siblings reared at 27°C (T_10_ = 3.02, p = 0.02) and marginally slower than 30°C (T_10_ = 2.49, p = 0.06).

In some arthropods, organisms much reach a minimum size before molting will begin [70]. We therefore examined the effects of temperature on spider mass and found that temperature had a significant effect on the size of spiders at the time of the second molt (Fig 5C; F_2,15_ = 4.284; *p* = 0.0337). While there was no significant difference between the predicted size for spiders housed at 27°C (4.37 ± 0.68 mg) and 30°C (3.99 ± 0.53 mg) at the time of the second molt, spiders reared at 33°C were significantly smaller (1.91 ± 0.67 mg) at the time of molt two than those reared at 27°C (T_10_ = 2.72, p = 0.04) and marginally smaller than those at 30°C (T_10_ = 2.30, p = 0.09; Fig 5C).

There was a significant effect of temperature on mortality (Fig 5D; F_2,15_ = 4.103; *p* = 0.0379). At 33°C, 11.0% ± 4.8% of spiders died during the course of the study, which was a significantly higher mortality rate than siblings reared at 27°C (T_10_ = -2.71, p = 0.04), of which only 4.3% ± 3.2% spiders did not survive the study (Fig 5D). No statistically significant differences in mortality rates were observed between spiders reared 30°C (5.6% ± 4.6%) when compared to those at 27°C, or 33°C.

## Discussion

As reported here and in Johnson et al. [7], despite the apparent success of *L*. *hesperus* in urban Phoenix, UHI conditions are correlated with decreased growth rates, delayed development, and increased mortality. We have found that these heat-induced developmental changes are associated with underlying changes in ecdysteroid titers, which respond to increased temperatures in two ways. First, slightly elevated temperatures, from 27 to 30˚C, result in a molting peak that is delayed and reduced in size, while increasing the temperature to UHI conditions (33˚C) appears to completely abolish the molting peak. The second ecdysteroid response to elevated temperatures occurs during the intermolt period between the second and third molts. During this time, raising the temperature to intermediate temperatures (30˚C) does not alter the basal ecdysteroid titers. However, the intermolt ecdysteroid titers are significantly elevated in response to UHI temperatures (33˚C).

Developmental timing (e.g. time between molts), which is regulated by ecdysteroids, is highly dependent upon temperature in arthropods [84]. Previous reports, as well as the present study, have indicated that there is an optimum temperature for development and temperatures that stray too far outside of this range have negative effects on arthropod growth, developmental times, and mortality [7, 85]. We have observed this response in *L*. *hesperus* and, although the spiders utilized in this study originated from urban populations, their optimum temperature for development appears to be 27˚C, the average temperature of spider microclimates in the non-urban Sonoran Desert [7]. At this temperature, a surge of ecdysteroids initiates molting, and only low levels of spider mortality is observed. As temperatures move away from this optimum, from 27 to 30˚C, the 20E surge is reduced and delayed, although these changes do not appear to have a significant effect on molting times, growth rates, or mortality. In contrast, raising temperatures to 33˚C, the average summer microclimate temperatures experienced by urban spiders, the ecdysteroid surge is reduced or missing and spiders exhibit significant molting delays, slower growth rates, and increased spider mortality. Although our results do not take into consideration daily fluctuations in temperature, a variable that must be addressed in future studies, our data suggest that 33˚C exceeds the temperature threshold for development and any spiders that are able survive to adulthood will be smaller than their counterparts in the non-urban desert.

In various arthropods, short-term exposure to temperatures above the optimum developmental threshold can lead to a delay in development, with recovery possible after returning to normal conditions [86–88]. However, UHI conditions are not temporary and studies with Sonoran *Drosophila* species suggest that, at least in this case, microhabitats provide little-to-no respite from extreme temperatures [89]. Results presented here suggest that temperatures associated with urban heat islands represent chronic thermal stress that disrupts the ecdysteroid surge that is essential for molting, resulting in overall negative effects on spider development. The possibility that a return to lower temperatures could restore the normal developmental profile, both in terms of ecdysteroid titers and developmental timing, is intriguing, but is outside the scope of the present study.

In many arthropods, formation of the molt-inducing ecdysteroid peak occurs in response to the attainment of a critical weight, a minimal mass which indicates that sufficient nutritional stores have been attained to support the upcoming molt [47, 70, 90]. While critical weight has not been reported in spiders, the presence of a size-sensing mechanism that triggers ecdysteroid production would further explain why the pre-molt ecdysteroid peak is delayed in spiders reared at high temperatures, which have significantly slower growth rates than their cooler counterparts.

A small increase in basal ecdysteroid levels has been reported in arthropods reared under stressful conditions including chronic intoxication, nutrient shortage, oxidative stress, sleep deprivation, high population density, injury, and thermal stress [77, 91–94]. A similar endocrine response to elevated temperatures has been studied extensively in *Drosophila melanogaster*, with an increase in ecdysteroid levels being seen within 60 minutes of exposure to heat stress and, while not attaining the same intensity of the morphogenic peak, intermolt hemolymph ecdysteroid titers are significantly elevated compared to non-stressed controls [77, 94].

Published reports suggest that elevated basal 20E titers may serve a protective role during stressful conditions, but only if the concentrations are below those that induce molting and metamorphosis [95–97]. Moderately elevated ecdysteroid titers are associated with increased resistance to formaldehyde in the blue bottle fly *Calliphora vicina* and in the silkworm *Bombyx mori* [95]. In fruit flies (*Drosophila melanogaster)* and the greater wax moth (*Galleria mellonella*), moderately elevated basal 20E titers are required for the regeneration of damaged tissues while high 20E concentrations induce developmental transitions (e.g. pupation) but suppress tissue regeneration [96, 97]. Protective effects of increased basal ecdysteroid titers have also been reported following bacterial infection, where moderate 20E titers reduce mortality associated with paralyzing toxins and septicemia induced by spore infection [91].

Elevated intermolt ecdysteroid concentrations induced in spiders exposed to extreme heat might serve a thermoprotective role by altering physiological and behavioral responses so that any animals that are able to survive to adulthood can better cope with the unfavorable urban environment. Increased tolerance to high temperatures in city-dwelling arthropods has been reported for urban leaf-cutter ants, which are more thermotolerant than their rural counterparts [98]. While ecdysteroid levels in heat tolerant ants has not been examined, ecdysteroid have been linked to thermotolerance in other systems [99, 100]. Ecdysteroids can induce expression of various genes encoding heat shock proteins (Hsps), even in the absence of heat [99, 100]. Heat shock proteins are chaperones that prevent the aggregation of denatured proteins and are associated with resistance to heat shock and other forms of stress [101, 102]. Enhanced expression of Hsps, specifically *Hsp30* and *Hsp70*, in desert species of the small freshwater fish *Poeciliopsis* is thought to confer thermal resistance, allowing the fish to survive elevated temperatures found in desert regions of northwest Mexico [103, 104]. Similarly, increased expression of Hsp genes including *Hsp90* and *Hsp47* confers thermal resistance to a population of minnows (*Puntius sopher*) found in hot spring run-offs in Odisha, India [105]. Increased thermotolerance has also been reported in cultured cells that overexpress *Hsp70* or *Hsp27* [106–109]. Although Hsps that are induced in response to thermal stress have been identified in the wolf spider, *Pardosa pseudoannulata*, ecdysteroid regulation of Hsps in spiders has not been established [110].

Elevated basal ecdysteroid titers might also mediate behavioral changes in web-building and aggression that have been reported in urban spiders [7]. Urban males show increased aggression towards prey when compared to males at lower non-urban desert temperatures while juvenile urban females have reduced web building behavior and produce smaller webs [7]. A link has been shown between ecydsteroid concentrations and web-building behavior in the orb-weaver spiders, *Cyclosa morretes* and *Cyclosa fililineata* [111]. These spiders serve as hosts for parasitoid wasps, which manipulate web-building behavior of their spider hosts by injecting ecdysteroids [111]. Parasitized orb-weaver spiders display elevated ecdysteroid titers and build ‘cocoon webs’ which are thought to protect the wasp’s cocoon as it develops [111]. A link between ecdysteroids and aggressive behavior has not been examined in spiders but has been demonstrated in other organisms. In honeybees, dominant workers had significant higher ecdysteroid titers than lower-ranked workers and presented with higher levels of aggression [112]. In American lobsters, *Homarus americanus*, elevated ecdysteroid titers have also been linked to increased aggression [113–115]. Pre-molt lobsters have high levels of 20E and are more aggressive than lobsters at different developmental stages [114]. In addition, lobsters injected with 20E also display an increase in aggressive behavior [115].

It should be noted that genomic work suggests large genetic variation between urban Phoenix and nearby Sonoran Desert black widow populations [116]. The present study has focused solely on responses in urban spiders; however, the possibility exists that spiders from desert lineages would respond differently to UHI temperatures. A natural extension of our work would be to compare ecdysteroid titers, development, and behavior in urban and desert spider lineages reared at both desert (27˚C) and urban (33˚C) temperatures. Indeed, we have recently shown an intriguing interaction in which desert lineages are more cannibalistic than urban ones, but the UHI temperature of 33˚C makes all spiderlings heighten cannibalism [117].

## Conclusion

Despite the apparent success of *L*. *hesperus* as an urban exploiter, we find that elevated urban temperatures significantly delay and reduce the ecdysteroid peak that precedes the second molt, a finding that is consistent with the developmental retardation observed in urban spiders [7]. Our studies suggest that current urban temperatures permit limited survival and moving forward, *L*. *hesperus* with less susceptibility to environmental extremes will likely be selected for as temperatures continue to rise. We also find that urban temperatures are associated with significantly elevated intermolt ecdysteroid titers which may, at least in part, counteract the negative effects on developmental progression. The potential thermoprotective effects of elevated basal ecdysteroids, ecdysteroid mediated changes in spider behavior, and other factors such as increased prey abundance [118] and temperature variation within a 24-hour period, may serve to counteract some of the negative consequences of increased temperatures, allowing black widows to continue exploiting urban settings.

## Supporting information

S1 Fig *L*. *hesperus* collection sites in the Phoenix metropolitan area. Samples were collected from the following sites FLW (Frank Lloyd Wright), MR (Marshall Ranch), SBF (Sun Burst Farms), OLI (Olive), AVOW (Avondale West—Wigwam Creek Middle School), and AVOC (Corte Sierra Middle School). Spider families were named based on collection site.
(TIF)

S2 Fig Variation in family ecdysteroid titers during spiderling development. (A) A significant effect of family on average ecdysteroid titers was observed at 27˚C (F_9,103_ = 4.13; p = 0.0001). (B) Most families had lower ecdysteroid titers at intermediate temperatures and higher ecdysteroid titers at urban temperatures when compared to titers observed at desert temperatures.
(TIF)

S1 Table Spider ecdysteroid and developmental measurements. (DOCX)

## References

[1] Martens WJM. Climate change, thermal stress and mortality changes. Soc Sci Med. 1998;46: 331–344. doi: 10.1016/s0277-9536(97)00162-7 9460815

[2] Zhang T, Huang Y, Yang X. Climate warming over the past three decades has shortened rice growth duration in China and cultivar shifts have further accelerated the process for late rice. Glob Chang Biol. 2013;19: 563–570. doi: 10.1111/gcb.12057 23504793

[3] Meehl GA, Tebaldi C. More intense, more frequent, and longer lasting heat waves in the 21st century. Science (80-). 2004;305: 994–997. doi: 10.1126/science.1098704 15310900

[4] Bagath M, Krishnan G, Devaraj C, Rashamol VP, Pragna P, Lees AM, et al. The impact of heat stress on the immune system in dairy cattle: A review. Research in Veterinary Science. Elsevier B.V.; 2019. pp. 94–102. doi: 10.1016/j.rvsc.2019.08.011 31445399

[5] Kirk Green C, Moore PJ, Sial AA. Impact of heat stress on development and fertility of Drosophila suzukii Matsumura (Diptera: Drosophilidae). J Insect Physiol. 2019;114: 45–52. doi: 10.1016/j.jinsphys.2019.02.008 30796949

[6] Romo-Barron CB, Diaz D, Portillo-Loera JJ, Romo-Rubio JA, Jimenez-Trejo F, Montero-Pardo A. Impact of heat stress on the reproductive performance and physiology of ewes: a systematic review and meta-analyses. Int J Biometeorol. 2019. doi: 10.1007/s00484-019-01707-z 30888508

[7] Johnson JC, Urcuyo J, Moen C, Stevens DR. Urban heat island conditions experienced by the Western black widow spider (Latrodectus hesperus): Extreme heat slows development but results in behavioral accommodations. Kuntner M, editor. PLoS One. 2019;14: e0220153. doi: 10.1371/journal.pone.0220153 31490963 PMC6730917

[8] Hall JM, Warner DA. Thermal spikes from the urban heat island increase mortality and alter physiology of lizard embryos. J Exp Biol. 2018;221. doi: 10.1242/jeb.181552 30021761

[9] Evans RK, Toews MD, Sial AA. Impact of short- and long-term heat stress on reproductive potential of Drosophila suzukii Matsumura (Diptera: Drosophilidae). J Therm Biol. 2018;78: 92–99. doi: 10.1016/j.jtherbio.2018.09.011 30509672

[10] Nelson KC, Palmer MA, Pizzuto JE, Moglen GE, Angermeier PL, Hilderbrand RH, et al. Forecasting the combined effects of urbanization and climate change on stream ecosystems: From impacts to management options. J Appl Ecol. 2009;46: 154–163. doi: 10.1111/j.1365-2664.2008.01599.x 19536343 PMC2695864

[11] Ratriyanto A, Mosenthin R. Osmoregulatory function of betaine in alleviating heat stress in poultry. J Anim Physiol Anim Nutr (Berl). 2018;102: 1634–1650. doi: 10.1111/jpn.12990 30238641

[12] Seress G, Hammer T, Bókony V, Vincze E, Preiszner B, Pipoly I, et al. Impact of urbanization on abundance and phenology of caterpillars and consequences for breeding in an insectivorous bird. Ecol Appl. 2018;28: 1143–1156. doi: 10.1002/eap.1730 29679462

[13] United Nations, Department of Economic and Social Affairs PD. World Urbanization Prospects: The 2018 Revision (ST/ESA/SER.A/420). Demogr Res. 2019;12: 197–236. Available: https://population.un.org/wup/Publications/Files/WUP2018-Report.pdf

[14] Pauchard A, Aguayo M, Peña E, Urrutia R. Multiple effects of urbanization on the biodiversity of developing countries: The case of a fast-growing metropolitan area (Concepción, Chile). Biol Conserv. 2006;127: 272–281. doi: 10.1016/j.biocon.2005.05.015

[15] Hawkins TW, Brazel AJ, Stefanov WL, Bigler W, Saffell EM, Hawkins TW, et al. The Role of Rural Variability in Urban Heat Island Determination for Phoenix, Arizona. J Appl Meteorol. 2004;43: 476–486. doi: 10.1175/1520-0450(2004)043&lt;0476:TRORVI&gt;2.0.CO;2

[16] Kim HH. Urban heat island. Int J Remote Sens. 1992;13: 2319–2336. doi: 10.1080/01431169208904271

[17] Isaksson C. Impact of Urbanization on Birds. In: Tietze DT, editor. Bird Species: How They Arise, Modify and Vanish. Cham: Springer International Publishing; 2018. pp. 235–257. doi: 10.1007/978-3-319-91689-7_13

[18] Shochat E, Lerman S, Fernández-Juricic E. Birds in Urban Ecosystems: Population Dynamics, Community Structure, Biodiversity, and Conservation. Urban Ecosystem Ecology. John Wiley & Sons, Ltd; 2015. pp. 75–86. doi: 10.2134/agronmonogr55.c4

[19] Denys C, Schmidt H. Insect communities on experimental mugwort (Artemisia vulgaris L.) plots along an urban gradient. Oecologia. 1998;113: 269–277. doi: 10.1007/s004420050378 28308207

[20] McIntyre N. Ecology of Urban Arthropods: A Review and a Call to Action. Ann Entomol Soc Am. 2009;93: 825–835. doi: 10.1603/0013-8746(2000)093[0825:EOUAAR]2.0.CO;2

[21] Zhao Z, Sharifi A, Dong X, Shen L, He BJ. Spatial Variability and Temporal Heterogeneity of Surface Urban Heat Island Patterns and the Suitability of Local Climate Zones for Land Surface Temperature Characterization. Remote Sens 2021, Vol 13, Page 4338. 2021;13: 4338. doi: 10.3390/RS13214338

[22] Yang J, Yang Y, Sun D, Jin C, Xiao X. Influence of urban morphological characteristics on thermal environment. Sustain Cities Soc. 2021;72: 103045. doi: 10.1016/J.SCS.2021.103045

[23] Yang J, Wang Y, Xue B, Li Y, Xiao X, Xia J(Cecilia), et al. Contribution of urban ventilation to the thermal environment and urban energy demand: Different climate background perspectives. Sci Total Environ. 2021;795: 148791. doi: 10.1016/j.scitotenv.2021.148791 34237531

[24] Luo X, Yang J, Sun W, He B. Suitability of human settlements in mountainous areas from the perspective of ventilation: A case study of the main urban area of Chongqing. J Clean Prod. 2021;310: 127467. doi: 10.1016/J.JCLEPRO.2021.127467

[25] Qiao Z, Liu L, Qin Y, Xu X, Wang B, Liu Z. The Impact of Urban Renewal on Land Surface Temperature Changes: A Case Study in the Main City of Guangzhou, China. Remote Sens 2020, Vol 12, Page 794. 2020;12: 794. doi: 10.3390/RS12050794

[26] Han D, Yang X, Cai H, Xu X. Impacts of Neighboring Buildings on the Cold Island Effect of Central Parks: A Case Study of Beijing, China. Sustain 2020, Vol 12, Page 9499. 2020;12: 9499. doi: 10.3390/SU12229499

[27] Blair RB. Land Use and Avian Species Diversity Along an Urban Gradient. Ecol Appl. 1996;6: 506–519. doi: 10.2307/2269387

[28] Goumas M, Boogert NJ, Kelley LA. Urban herring gulls use human behavioural cues to locate food. R Soc Open Sci. 2020;7. doi: 10.1098/rsos.191959 32257348 PMC7062050

[29] Ferman LM, Peter H-U, Montalti D. A study of feral pigeon Columba livia var. in urban and suburban areas in the city of Jena, Germany. Arx Miscel·lània Zoològica. 2010. Available: http://www.bcn.cat/mciencies/publicacions/AMZ/vol8.1/AMZ-1001.htm

[30] Sacchi R, Gentilli A, Razzetti E, Barbieri F. Effects of building features on density and flock distribution of feral pigeons Columba livia var. domestica in an urban environment. Can J Zool. 2002;80: 48–54. doi: 10.1139/z01-202

[31] Luniak M. Synurbization-adaptation of animal wildlife to urban development. 4th International Urban Wildlife Symposium. 2004. pp. 50–55. Available: https://ci.nii.ac.jp/naid/10027356431/

[32] Buijs JA, Van Wijnen JH. Survey of feral rock doves (Columba livia) in Amsterdam, a bird-human association. Urban Ecosyst. 2001;5: 235–241. doi: 10.1023/A:1025667127518

[33] Garber SD. The Urban Naturalist. Somerset, New Jersey, USA: John Wiley & Sons Inc; 1987. Available: https://books.google.com/books?id=NlLwAAAAMAAJ

[34] Douglas I, James P. Urban ecology: an introduction. London and New York: Routledge, Taylor and Francis Group; 2015. Available: http://ezproxy.deakin.edu.au/login?url=https://ebookcentral.proquest.com/lib/deakin/detail.action?docID=1829291

[35] Bang C, Faeth SH, Sabo JL. Control of arthropod abundance, richness, and composition in a heterogeneous desert city. Ecol Monogr. 2012;82: 85–100. doi: 10.1890/11-0828.1

[36] McKinney ML. Urbanization, Biodiversity, and Conservation. Bioscience. 2002;52: 883. doi: 10.1641/0006-3568(2002)052[0883:UBAC]2.0.CO;2

[37] Kaiser A, Merckx T, Van Dyck H. The Urban Heat Island and its spatial scale dependent impact on survival and development in butterflies of different thermal sensitivity. Ecol Evol. 2016;6: 4129–4140. doi: 10.1002/ece3.2166 27516869 PMC4972237

[38] Lowe EC, Wilder SM, Hochuli DF. Urbanisation at Multiple Scales Is Associated with Larger Size and Higher Fecundity of an Orb-Weaving Spider. Chapman M (Gee) G, editor. PLoS One. 2014;9: e105480. doi: 10.1371/journal.pone.0105480 25140809 PMC4139358

[39] Buckley LB, Huey RB. Temperature extremes: geographic patterns, recent changes, and implications for organismal vulnerabilities. Glob Chang Biol. 2016;22: 3829–3842. doi: 10.1111/gcb.13313 27062158

[40] Trubl P, Gburek T, Miles L, Johnson JC. Black widow spiders in an urban desert: Population variation in an arthropod pest across metropolitan Phoenix, AZ. Urban Ecosyst. 2012;15: 599–609. doi: 10.1007/s11252-011-0223-2

[41] Johnson J, Garver E, Martin T. Black widows on an urban heat island: extreme heat affects spider development and behavior from egg to adulthood. Anim Behav. 2020;167: 77–84. 10.1016/j.anbehav.2020.07.005

[42] Ashburner M. Patterns of puffing activity in the salivary gland chromosomes of Drosophila. VI. Induction by ecdysone in salivary glands of D. melanogaster cultured in vitro. Chromosoma. 1972;38: 255–81. doi: 10.1007/BF00290925 4627363

[43] Kiss I, Molnár I. Metamorphic changes of wild type and mutant Drosophila tissues induced by 20-hydroxy ecdysone in vitro. J Insect Physiol. 1980;26: 391–401. doi: 10.1016/0022-1910(80)90010-4

[44] Krishnakumaran A, Schneiderman HA. Control of molting in mandibulate and chelicerate arthropods by ecdysones. Biol Bull. 1970;139: 520–538. doi: 10.2307/1540371 5494238

[45] Mandaron P, Guillerment C, Sengel P. In Vitro Development of Drosophila Imaginal Discs: Hormonal Control and Mechanism of Evagination. Integr Comp Biol. 1977;17: 661–670. doi: 10.1093/icb/17.3.661

[46] Nijhout HF, Callier V. Developmental Mechanisms of Body Size and Wing-Body Scaling in Insects. Annu Rev Entomol. 2015;60: 141–156. doi: 10.1146/annurev-ento-010814-020841 25341104

[47] Nijhout HF, Williams CM. Control of Moulting and Metamorphosis in the Tobacco Hornworm, Manduca Sexta (L.): Cessation of Juvenile Hormone Secretion as a Trigger for Pupation. J Exp Biol. 1974;61: 493–501. Available: doi: 10.1242/jeb.61.2.493 4443741

[48] Postlethwait JH, Schneiderman HA. Induction of metamorphosis by ecdysone analogues. Drosophila imaginal discs cultured in vivo. Biol Bull. 1970;138: 47–55. Available: http://www.ncbi.nlm.nih.gov/pubmed/5437915 doi: 10.2307/1540290 5437915

[49] Yamanaka N, Rewitz KF, O’Connor MB. Ecdysone Control of Developmental Transitions: Lessons from *Drosophila* Research. Annu Rev Entomol. 2013;58: 497–516. doi: 10.1146/annurev-ento-120811-153608 23072462 PMC4060523

[50] Calvez B, Hirn M, De Reggi M. Ecdysone changes in the haemolymph to two silkworms (Bombyx mori and Philosamia cynthia) during larval and pupal development. FEBS Lett. 1976;72: 57–61. Available: http://www.ncbi.nlm.nih.gov/pubmed/992091 doi: 10.1016/0014-5793(76)80898-8 992091

[51] Hanaoka K, Onishi E. Changes in ecdysone titre during pupal-adult development in the silkworm, Bombyx mori. J Insect Physiol. 1974;20: 2375–84. Available: http://www.ncbi.nlm.nih.gov/pubmed/4436582 doi: 10.1016/0022-1910(74)90024-9 4436582

[52] Kunkel JG. Cockroach molting. II. The nature of regeneration-induced delay of molting hormone secretion. Biol Bull. 1977;153: 145–62. Available: http://www.ncbi.nlm.nih.gov/pubmed/889943 doi: 10.2307/1540698 889943

[53] Riddiford LM. Hormone receptors and the regulation of insect metamorphosis. Receptor. 1993;3: 203–9. Available: http://www.ncbi.nlm.nih.gov/pubmed/8167571 8167571

[54] Safranek L, Williams CM. Determinants of Larval Molt Initation in the Tobacco Hornworm, Manduca Sexta. Biol Bull. 1984;167: 568–578. doi: 10.2307/1541410 29320264

[55] Sakurai S, Warren JT, Gilbert LI. Ecdysteroid synthesis and molting by the tobacco hornworm,Manduca sexta, in the absence of prothoracic glands. Arch Insect Biochem Physiol. 1991;18: 13–36. doi: 10.1002/arch.940180103 1932778

[56] Sonobe H, Yamada R. Ecdysteroids during Early Embryonic Development in Silkworm Bombyx mori: Metabolism and Functions. Zoolog Sci. 2004;21: 503–516. doi: 10.2108/zsj.21.503 15170054

[57] Bonaric JC, De Reggi M. Changes in ecdysone levels in the spiderPisaura mirabilis nymphs (Araneae, Pisauridae). Experientia. 1977;33: 1664–1665. doi: 10.1007/BF01934060

[58] Trabalon M, Pourié G, Hartmann N. Relationships among cannibalism, contact signals, ovarian development and ecdysteroid levels in Tegenaria atrica (Araneae, Agelenidae). Insect Biochem Mol Biol. 1998;28: 751–758. doi: 10.1016/S0965-1748(98)00066-6

[59] Trabalon M, Bautz AM, Moriniere M, Porcheron P. Ovarian development and correlated changes in hemolymphatic ecdysteroid levels in two spiders, Coelotes terrestris and Tegenaria domestica (Araneae, Agelenidae). Gen Comp Endocrinol. 1992;88: 128–36. Available: http://www.ncbi.nlm.nih.gov/pubmed/1426956 doi: 10.1016/0016-6480(92)90201-t 1426956

[60] Trabalon M, Blais C. Juvenile Development, Ecdysteroids and Hemolymph Level of Metabolites in the Spider Brachypelma albopilosum (Theraphosidae). J Exp Zool Part A Ecol Genet Physiol. 2012;317: 236–247. doi: 10.1002/jez.1717 22311802

[61] Honda Y, Ishiguro W, Ogihara MH, Kataoka H, Taylor D. Identification and expression of nuclear receptor genes and ecdysteroid titers during nymphal development in the spider Agelena silvatica. Gen Comp Endocrinol. 2017;247: 183–198. doi: 10.1016/j.ygcen.2017.01.032 28174130

[62] Beldade P, Mateus ARA, Keller RA. Evolution and molecular mechanisms of adaptive developmental plasticity. Mol Ecol. 2011;20: 1347–1363. doi: 10.1111/j.1365-294X.2011.05016.x 21342300

[63] Brakefield PM, Zwaan BJ. Seasonal polyphenisms and environmentally induced plasticity in the Lepidoptera: The coordinated evolution of many traits on multiple levels. Oxford University Press; 2011. Available: https://research.wur.nl/en/publications/seasonal-polyphenisms-and-environmentally-induced-plasticity-in-t

[64] Sommer RJ, Ogawa A. Hormone Signaling and Phenotypic Plasticity in Nematode Development and Evolution. Curr Biol. 2011;21: R758–R766. doi: 10.1016/j.cub.2011.06.034 21959166

[65] Zera AJ, Harshman LG. The Physiology of Life History Trade-Offs in Animals. Annu Rev Ecol Syst. 2001;32: 95–126. doi: 10.1146/annurev.ecolsys.32.081501.114006

[66] Brisson JA. Aphid wing dimorphisms: linking environmental and genetic control of trait variation. Philos Trans R Soc B Biol Sci. 2010;365: 605–616. doi: 10.1098/rstb.2009.0255 20083636 PMC2817143

[67] Dawson A. Control of the annual cycle in birds: endocrine constraints and plasticity in response to ecological variability. Philos Trans R Soc B Biol Sci. 2008;363: 1621–1633. doi: 10.1098/rstb.2007.0004 18048294 PMC2606722

[68] Denlinger DL. Regulation of Diapause. Annu Rev Entomol. 2002;47: 93–122. doi: 10.1146/annurev.ento.47.091201.145137 11729070

[69] Emlen DJ, Warren IA, Johns A, Dworkin I, Lavine LC. A Mechanism of Extreme Growth and Reliable Signaling in Sexually Selected Ornaments and Weapons. Science (80-). 2012;337: 860–864. doi: 10.1126/science.1224286 22837386

[70] Davidowitz G, D’Amico LJ, Nijhout HF. Critical weight in the development of insect body size. Evol Dev. 2003;5: 188–97. Available: http://www.ncbi.nlm.nih.gov/pubmed/12622736 doi: 10.1046/j.1525-142x.2003.03026.x 12622736

[71] Oostra V, Mateus ARA, van der Burg KRL, Piessens T, van Eijk M, Brakefield PM, et al. Ecdysteroid hormones link the juvenile environment to alternative adult life histories in a seasonal insect. Am Nat. 2014;184: E79–92. doi: 10.1086/677260 25141151

[72] Simpson SJ, Sword GA, Lo N. Polyphenism in Insects. Curr Biol. 2011;21: R738–R749. doi: 10.1016/j.cub.2011.06.006 21959164

[73] Simpson SJ, Sword GA. Phase polyphenism in locusts: mechanisms, population consequences, adaptive significance and evolution. Phenotypic Plast insects Mech consequences. 2009; 147–189. Available: https://www.cabdirect.org/cabdirect/abstract/20123375437

[74] Denver RJ. Environmental Stress as a Developmental Cue: Corticotropin-Releasing Hormone Is a Proximate Mediator of Adaptive Phenotypic Plasticity in Amphibian Metamorphosis. Horm Behav. 1997;31: 169–179. doi: 10.1006/hbeh.1997.1383 9154437

[75] Bownes M. The roles of juvenile hormone, ecdysone and the ovary in the control of Drosophila vitellogenesis. J Insect Physiol. 1989;35: 409–413. doi: 10.1016/0022-1910(89)90115-7

[76] Gruntenko NE, Bownes M, Terashima J, Sukhanova MZ, Raushenbach IY. Heat stress affects oogenesis differently in wild-type Drosophila virilis and a mutant with altered juvenile hormone and 20-hydroxyecdysone levels. Insect Mol Biol. 2003;12: 393–404. doi: 10.1046/j.1365-2583.2003.00424.x 12864919

[77] Meiselman MR, Kingan TG, Adams ME. Stress-induced reproductive arrest in Drosophila occurs through ETH deficiency-mediated suppression of oogenesis and ovulation. BMC Biol. 2018;16: 18. doi: 10.1186/s12915-018-0484-9 29382341 PMC5791332

[78] Rauschenbach IY, Sukhanova MZ, Hirashima A, Sutsugu E, Kuano E. Role of the Ecdysteroid System in the Regulation of Drosophila Reproduction under Environmental Stress. Dokl Biol Sci. 2000;375: 641–643. doi: 10.1023/a:1026610425973 11214597

[79] Terashima J, Takaki K, Sakurai S, Bownes M. Nutritional status affects 20-hydroxyecdysone concentration and progression of oogenesis in Drosophila melanogaster. J Endocrinol. 2005;187: 69–79. doi: 10.1677/joe.1.06220 16214942

[80] Soller M, Bownes M, Kubli E. Control of oocyte maturation in sexually mature Drosophila females. Dev Biol. 1999;208: 337–351. doi: 10.1006/dbio.1999.9210 10191049

[81] Gruntenko NE, Karpova EK, Alekseev AA, Faddeeva N V., Raushenbakh IY. Experimental decrease in dopamine level dramatically decreases Drosophila virilis fitness. Dokl Biol Sci. 2005;401: 127–129. doi: 10.1007/s10630-005-0063-4 16003876

[82] Michaud S, Tanguay RM. Expression of the Hsp23 chaperone during Drosophila embryogenesis: Association to distinct neural and glial lineages. BMC Dev Biol. 2003;3: 1–12. doi: 10.1186/1471-213x-3-1 14617383 PMC293469

[83] Chang W, Cheng J, Allaire J, Xie Y, McPherson J. Shiny: web application framework for R. In: R Package. 2017.

[84] Nylin S, Gotthard K. Plasticity in Life-History Traits. Annu Rev Entomol. 1998;43: 63–83. doi: 10.1146/annurev.ento.43.1.63 9444750

[85] Govindan BN, Hutchison WD. Influence of Temperature on Age-Stage, Two-Sex Life Tables for a Minnesota-Acclimated Population of the Brown Marmorated Stink Bug (Halyomorpha halys). Insects. 2020;11: 108. doi: 10.3390/insects11020108 32046093 PMC7073653

[86] Davis JA, Radcliffe EB, Ragsdale DW. Effects of High and Fluctuating Temperatures on Myzus persicae (Hemiptera: Aphididae). Environ Entomol. 2006;35: 1461–1468. doi: 10.1093/ee/35.6.1461

[87] Asin L, Pons X. Effect of High Temperature on the Growth and Reproduction of Corn Aphids (Homoptera: Aphididae) and Implications for Their Population Dynamics on the Northeastern Iberian Peninsula. Environ Entomol. 2001;30: 1127–1134. doi: 10.1603/0046-225x-30.6.1127

[88] Nowierski RM, Gutierrez AP, Yaninek JS. Estimation of Thermal Thresholds and Age-Specific Life Table Parameters for the Walnut Aphid (Homoptera: Aphididae) Under Field Conditions. Environ Entomol. 1983;12: 680–686. doi: 10.1093/ee/12.3.680

[89] Gibbs AG, Perkins MC, Markow TA. No place to hide: Microclimates of Sonoran Desert Drosophila. J Therm Biol. 2003;28: 353–362. doi: 10.1016/S0306-4565(03)00011-1

[90] Callier V, Nijhout HF. Control of body size by oxygen supply reveals size-dependent and size-independent mechanisms of molting and metamorphosis. Proc Natl Acad Sci U S A. 2011;108: 14664–14669. doi: 10.1073/pnas.1106556108 21873228 PMC3167549

[91] Chernysh SI. Neuroendocrine System in Insect Stress. In: Ivanović J, Janković-Hladni M, editors. Hormones and Metabolism in Insect Stress. CRC Press; 1991. pp. 69–97. doi: 10.1201/9781351073233-6

[92] Ishimoto H, Sakai T, Kitamoto T. Ecdysone signaling regulates the formation of long-term courtship memory in adult Drosophila melanogaster. Proc Natl Acad Sci. 2009;106: 6381–6386. doi: 10.1073/pnas.0810213106 19342482 PMC2669368

[93] Karlson P, Shaaya E. Der Ecdysontiter während der Insektenentwicklung-I. Eine Methode zur Bestimmung des Ecdysongehalts. J Insect Physiol. 1964;10: 797–804. doi: 10.1016/0022-1910(64)90060-5

[94] Hirashima A, Rauschenbach IY, Sukhanova MJ. Ecdysteroids in Stress Responsive and Nonresponsive Drosophila Lines under Stress Conditions. 2000; 2657–2662. doi: 10.1271/bbb.64.2657 11210130

[95] Chernysh SI, Lukhtanov VA, Simonenko NP. Adaptation to damage in the silkworm Bombyx mori L. (Lepidoptera, Bombycidae). II. Effects of ecdysterone and other adaptogens on larval resistance to entobacterin. Entomological review (USA). 1983. pp. 1–8.

[96] Madhavan K, Schneiderman H. Hormonal Control of Imaginal Disc Regeneration in Galleria Mellonella (Lepidoptera). Biol Bull. 1969;137. Available: http://www.biolbull.org/content/137/2/321.abstract

[97] Smith-Bolton RK, Worley MI, Kanda H, Hariharan IK. Regenerative growth in Drosophila imaginal discs is regulated by Wingless and Myc. Dev Cell. 2009;16: 797–809. doi: 10.1016/j.devcel.2009.04.015 19531351 PMC2705171

[98] Angilletta MJ, Wilson RS, Niehaus AC, Sears MW, Navas CA, Ribeiro PL. Urban Physiology: City Ants Possess High Heat Tolerance. Chown S, editor. PLoS One. 2007;2: e258. doi: 10.1371/journal.pone.0000258 17327918 PMC1797824

[99] Chang ES, Chang SA, Keller R, Reddy PS, Snyder MJ, Spees JL. Quantification of Stress in Lobsters: Crustacean Hyperglycemic Hormone, Stress Proteins, and Gene Expression 1. Amer Zool. 1999;39: 487–495. Available: https://academic.oup.com/icb/article-abstract/39/3/487/148420

[100] Thomas SR, Lengyel JA. Ecdysteroid-regulated heat-shock gene expression during Drosophila melanogaster development. Dev Biol. 1986;115: 434–438. doi: 10.1016/0012-1606(86)90263-0 3086161

[101] Haslbeck M, Vierling E. A First Line of Stress Defense: Small Heat Shock Proteins and Their Function in Protein Homeostasis Evolution of sHsps. J Mol Biol. 2015;427: 1537–1548. doi: 10.1016/j.jmb.2015.02.002 25681016 PMC4360138

[102] Perdrizet GA, Rewinski MJ, Noonan EJ, Hightower LE. Biology of the Heat Shock Response and Stress Conditioning. Cell Stress Proteins. Springer New York; 2009. pp. 7–35. doi: 10.1007/978-0-387-39717-7_2

[103] Norris C, DiIorio P, Schultz R, Hightower L. Variation in heat shock proteins within tropical and desert species of poeciliid fishes. Mol Biol Evol. 1995;12. doi: 10.1093/oxfordjournals.molbev.a040280 8524039

[104] Dilorio PJ, Holsinger K, Schultz RJ, Hightower LE. Quantitative Evidence That Both Hsc70 and Hsp70 Contribute to Thermal Adaptation in Hybrids of the Livebearing Fishes Poeciliopsis. Cell Stress & Chaperones. Cell Stress Society InternationalSpringer; 1996. pp. 139–147. doi: 10.2307/1601912 9222599 PMC248466

[105] Mahanty A, Purohit GK, Yadav RP, Mohanty S, Mohanty BP. hsp90 and hsp47 appear to play an important role in minnow Puntius sophore for surviving in the hot spring run-off aquatic ecosystem. Fish Physiol Biochem. 2017;43: 89–102. doi: 10.1007/s10695-016-0270-y 27522494

[106] Parsell D, Taulien J, Lindquist S. The role of heat-shock proteins in thermotolerance. Philos Trans R Soc London Ser B Biol Sci. 1993;339: 279–286. doi: 10.1098/rstb.1993.0026 8098532

[107] Li GC, Nussenzweig A. Thermotolerance and heat shock proteins: possible involvement of Ku autoantigen in regulating Hsp70 expression. EXS. Birkhäuser Basel; 1996. pp. 425–449. doi: 10.1007/978-3-0348-9088-5_29 8856989

[108] Landry J, Chretien P, Lambert H, Hickey E, Weber LA. Heat shock resistance conferred by expression of the human HSP27 gene in rodent cells. J Cell Biol. 1989;109: 7–15. doi: 10.1083/jcb.109.1.7 2745558 PMC2115456

[109] Laszlo A, Lit GC. Heat-resistant variants of Chinese hamster fibroblasts altered in expression of heat shock protein (thermal resistance/cell survival/70-kDa heat shock protein). Proc NatI Acad Sci USA. 1985. 10.1073/pnas.82.23.8029 PMC391435 3865213

[110] Xiao R, Wang L, Cao Y, Zhang G. Transcriptome response to temperature stress in the wolf spider Pardosa pseudoannulata (Araneae: Lycosidae). Ecol Evol. 2016;6: 3540–3554. doi: 10.1002/ece3.2142 27127612 PMC4842027

[111] Kloss TG, Gonzaga MO, De Oliveira LL, Sperber CF. Proximate mechanism of behavioral manipulation of an orb-weaver spider host by a parasitoid wasp. PLoS One. 2017;12. doi: 10.1371/journal.pone.0171336 28158280 PMC5291528

[112] Bloch G, Hefetz A, Hartfelder K. Ecdysteroid titer, ovary status, and dominance in adult worker and queen bumble bees (Bombus terrestris). J Insect Physiol. 2000;46: 1033–1040. doi: 10.1016/s0022-1910(99)00214-0 10802116

[113] Kravitz EA. Serotonin and aggression: Insights gained from a lobster model system and speculations on the role of amine neurons in a complex behavior. Journal of Comparative Physiology—A Sensory, Neural, and Behavioral Physiology. Springer Verlag; 2000. pp. 221–238. doi: 10.1007/s003590050423 10757238

[114] Coglianese DL, Cromarty SI, Kass-Simon G. Perception of the steroid hormone 20-hydroxyecdysone modulates agonistic interactions in Homarus americanus. Anim Behav. 2008;75: 2023–2034. doi: 10.1016/j.anbehav.2007.11.010 22940528

[115] Sipala MW. Hormonal and Pheromonal Effects of 20-Hydroxyecdysone in the American Lobster, Homarus americanus. University of Rhode Island. 2012. doi: 10.1016/j.yhbeh.2012.07.013

[116] Miles LS, Dyer RJ, Verrelli BC. Urban hubs of connectivity: contrasting patterns of gene flow within and among cities in the western black widow spider. Proc R Soc B Biol Sci. 2018;285: 20181224. doi: 10.1098/rspb.2018.1224 30068686 PMC6111156

[117] de Tranaltes C, Dunn J, Martin JM, Johnson JC. Siblicide in the city: the urban heat island accelerates sibling cannibalism in the black widow spider (Latrodectus hesperus). Urban Ecosyst 2021. 2021;1: 1–8. doi: 10.1007/S11252-021-01148-W

[118] Johnson JC, Trubl PJ, Miles LS. Black Widows in an Urban Desert: City-Living Compromises Spider Fecundity and Egg Investment Despite Urban Prey Abundance. Am Midl Nat. 2012;168: 333–340. doi: 10.1674/0003-0031-168.2.333

