# Supplementary figures and images for: Ecdysteroid responses to urban heat island conditions during development of the western black widow spider (Latrodectus hesperus)
Source: PLoS One. 2022 Apr 28;17(4):e0267398. doi: 10.1371/journal.pone.0267398 (PMC9049550; doi:10.1371/journal.pone.0267398)

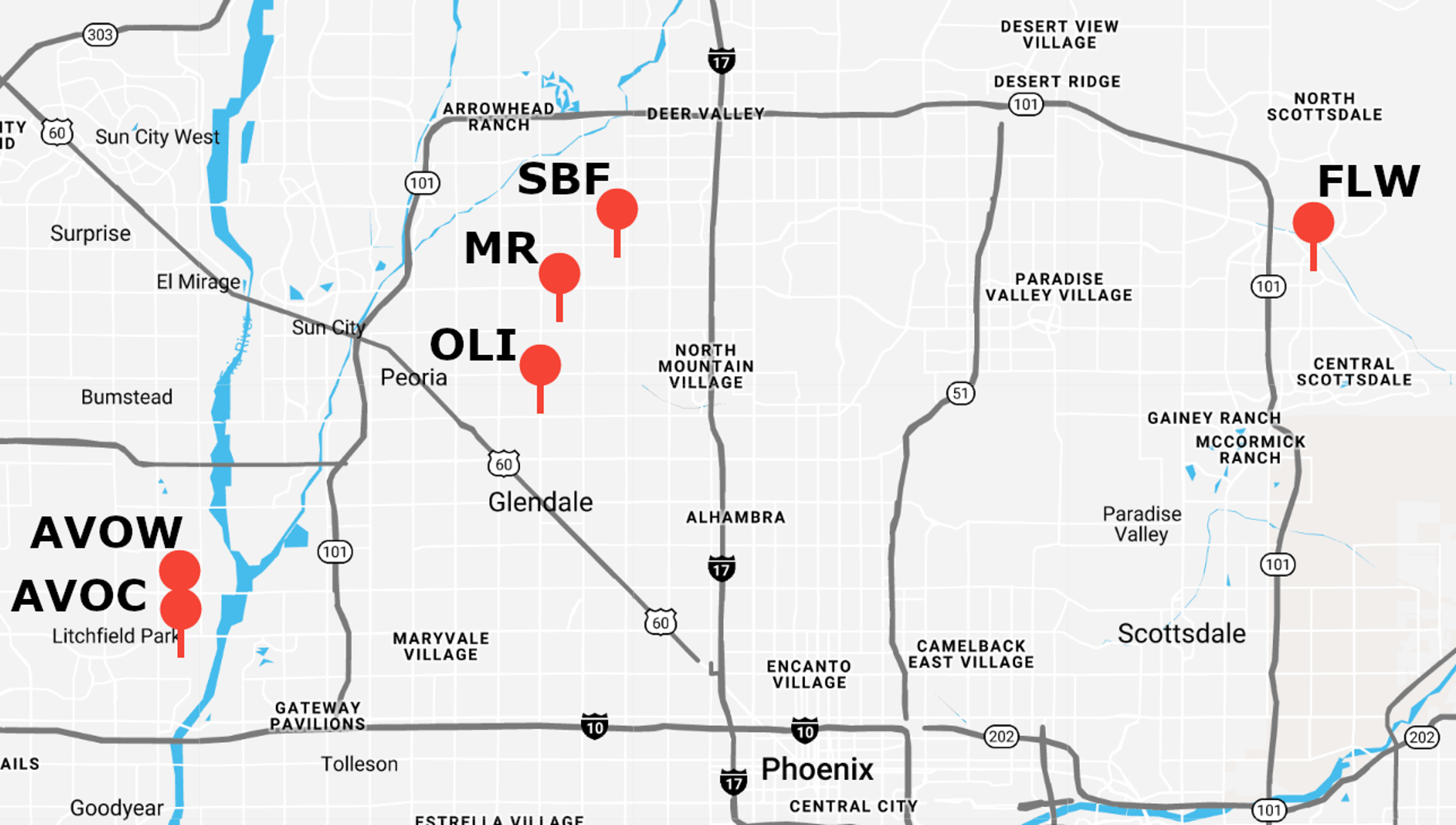

Supplement: S1 Fig — Samples were collected from the following sites FLW (Frank Lloyd Wright), MR (Marshall Ranch), SBF (Sun Burst Farms), OLI (Olive), AVOW (Avondale West—Wigwam Creek Middle School), and AVOC (Corte Sierra Middle School). Spider families were named based on collection site. (TIF) [file pone.0267398.s001.tif]

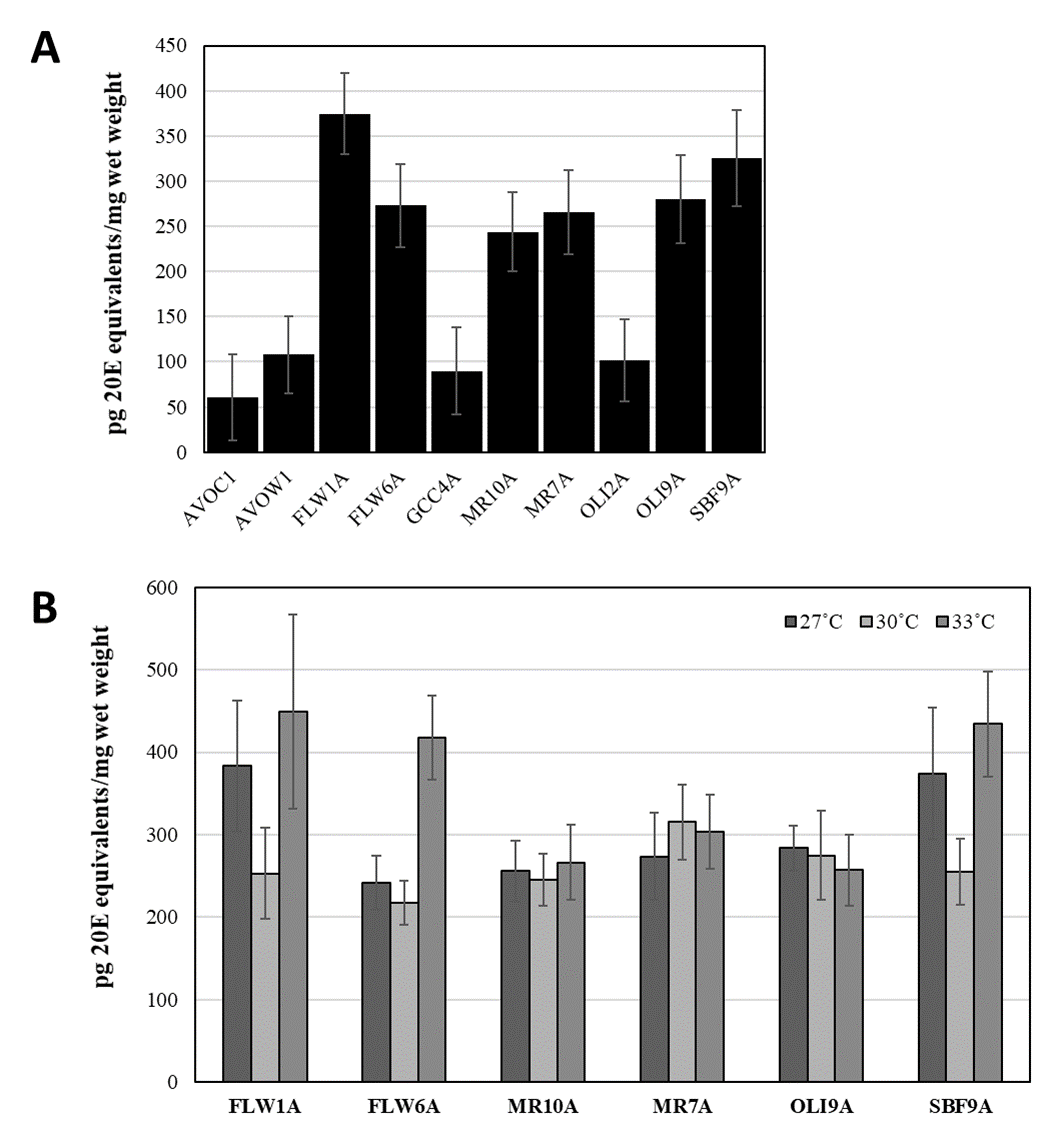

Supplement: S2 Fig — (A) A significant effect of family on average ecdysteroid titers was observed at 27˚C (F9,103 = 4.13; p = 0.0001). (B) Most families had lower ecdysteroid titers at intermediate temperatures and higher ecdysteroid titers at urban temperatures when compared to titers observed at desert temperatures. (TIF) [file pone.0267398.s002.tif]
