# Supplementary material for: Ecdysteroid responses to urban heat island conditions during development of the western black widow spider (Latrodectus hesperus)
Source: PLoS One. 2022 Apr 28;17(4):e0267398. doi: 10.1371/journal.pone.0267398 (PMC9049550; doi:10.1371/journal.pone.0267398)
Supplement: S1 Table — (DOCX) [file pone.0267398.s003.docx]

| Family | SpiderID | Average Time to Molt 2 by Family (Days) | Age (Days) | spider mass (mg) | pg 20E/spider | pg 20E/mg tissue | Rearing Temperature |
| --- | --- | --- | --- | --- | --- | --- | --- |
| FLW1A | FLW1A10 | 57 | 55 | 1.5 | 1357.42 | 904.95 | 27C |
| FLW1A | FLW1A17 | 57 | 56 | 1.1 | 164.13 | 149.21 | 27C |
| FLW1A | FLW1A15 | 57 | 57 | 1.4 | 394.07 | 281.48 | 27C |
| FLW1A | FLW1A19 | 57 | 58 | 1.6 | 1500.05 | 937.53 | 27C |
| FLW1A | FLW1A9 | 57 | 59 | 2.1 | 699.94 | 333.31 | 27C |
| FLW1A | FLW1A24 | 57 | 60 | 1.9 | 434.28 | 228.57 | 27C |
| FLW1A | FLW1A39 | 57 | 61 | 2.2 | 346.31 | 157.41 | 27C |
| FLW1A | FLW1A42 | 57 | 62 | 1.5 | 707.92 | 471.95 | 27C |
| FLW1A | FLW1A48 | 57 | 63 | 2.1 | 579.18 | 275.80 | 27C |
| FLW1A | FLW1A38 | 57 | 64 | 1.8 | 1289.33 | 716.30 | 27C |
| FLW1A | FLW1A52 | 57 | 65 | 1.3 | 291.12 | 223.94 | 27C |
| FLW1A | FLW1A56 | 57 | 66 | 1.1 | 193.46 | 175.87 | 27C |
| FLW1A | FLW1A61 | 57 | 67 | 3.2 | 404.82 | 126.51 | 27C |
| FLW1A | FLW1A85 | 57 | 68 | 2.9 | 396.79 | 136.83 | 27C |
| FLW1A | FLW1A87 | 57 | 69 | 3.1 | 531.75 | 171.53 | 27C |
| FLW1A | FLW1A89 | 57 | 70 | 2.3 | 1277.20 | 555.30 | 27C |
| FLW1A | FLW1A207 | 57 | 71 | 3.2 | 1277.20 | 399.13 | 27C |
| FLW1A | FLW1A90 | 57 | 72 | 2.2 | 1740.08 | 790.94 | 27C |
| FLW1A | FLW1A63 | 57 | 73 | 3 | 291.12 | 97.04 | 27C |
| FLW6A | FLW6A4 | 56 | 55 | 1.1 | 214.19 | 194.72 | 27C |
| FLW6A | FLW6A23 | 56 | 56 | 1 | 306.33 | 306.33 | 27C |
| FLW6A | FLW6A25 | 56 | 57 | 1.2 | 488.85 | 407.38 | 27C |
| FLW6A | FLW6A26 | 56 | 58 | 1.5 | 272.62 | 108.90 | 27C |
| FLW6A | FLW6A21 | 56 | 59 | 1.4 | 534.25 | 381.60 | 27C |
| FLW6A | FLW6A8 | 56 | 60 | 1.3 | 167.84 | 129.11 | 27C |
| FLW6A | FLW6A2 | 56 | 61 | 2.5 | 503.51 | 201.40 | 27C |
| FLW6A | FLW6A15 | 56 | 62 | 2.7 | 1004.93 | 372.20 | 27C |
| FLW6A | FLW6A9 | 56 | 63 | 2.3 | 220.93 | 96.06 | 27C |
| FLW6A | FLW6A30 | 56 | 64 | 1.6 | 239.05 | 149.41 | 27C |
| FLW6A | FLW6A40 | 56 | 65 | 1.8 | 601.95 | 334.42 | 27C |
| FLW6A | FLW6A52 | 56 | 66 | 1.6 | 346.92 | 216.82 | 27C |
| FLW6A | FLW6A64 | 56 | 67 | 1.4 | 502.38 | 358.84 | 27C |
| FLW6A | FLW6A245 | 56 | 68 | 1.5 | 1239.08 | 826.05 | 27C |
| FLW6A | FLW6A45 | 56 | 69 | 2 | 1166.57 | 583.28 | 27C |
| FLW6A | FLW6A37 | 56 | 70 | 2.4 | 423.40 | 176.42 | 27C |
| FLW6A | FLW6A48 | 56 | 71 | 2.9 | 423.18 | 145.92 | 27C |
| MR10A | MR10A9 | 60 | 55 | 1.2 | 280.19 | 233.49 | 27C |
| MR10A | MR10A16 | 60 | 56 | 0.9 | 265.81 | 295.35 | 27C |
| MR10A | MR10A5 | 60 | 57 | 1.9 | 518.63 | 272.96 | 27C |
| MR10A | MR10A33 | 60 | 58 | 1.5 | 867.62 | 578.41 | 27C |
| MR10A | MR10A36 | 60 | 59 | 1.3 | 183.42 | 141.10 | 27C |
| MR10A | MR10A24 | 60 | 60 | 2.1 | 173.27 | 82.51 | 27C |
| MR10A | MR10A40 | 60 | 61 | 1.6 | 382.09 | 238.81 | 27C |
| MR10A | MR10A8 | 60 | 62 | 1.8 | 334.03 | 185.57 | 27C |
| MR10A | MR10A25 | 60 | 63 | 2.7 | 498.15 | 184.50 | 27C |
| MR10A | MR10A50 | 60 | 64 | 2.5 | 446.94 | 178.78 | 27C |
| MR10A | MR10A41 | 60 | 65 | 2 | 1053.65 | 526.82 | 27C |
| MR10A | MR10A243 | 60 | 66 | 2.2 | 636.96 | 289.53 | 27C |
| MR10A | MR10A73 | 60 | 67 | 2 | 204.26 | 102.13 | 27C |
| MR10A | MR10A79 | 60 | 68 | 2.5 | 654.72 | 261.89 | 27C |
| MR10A | MR10A101 | 60 | 69 | 1.9 | 841.17 | 442.72 | 27C |
| MR10A | MR10A249 | 60 | 70 | 2.6 | 198.79 | 76.46 | 27C |
| MR10A | MR10A6 | 60 | 71 | 1.5 | 567.01 | 378.01 | 27C |
| MR10A | MR10A80 | 60 | 72 | 1.7 | 639.23 | 376.02 | 27C |
| MR10A | MR10A78 | 60 | 73 | 1.3 | 233.89 | 179.92 | 27C |
| MR10A | MR10A14 | 60 | 74 | 2.1 | 390.44 | 185.92 | 27C |
| MR10A | MR10A65 | 60 | 75 | 1.7 | 214.19 | 125.99 | 27C |
| MR7A | MR7A12 | 56 | 55 | 1.2 | 204.28 | 170.24 | 27C |
| MR7A | MR7A49 | 56 | 56 | 1.5 | 283.44 | 188.96 | 27C |
| MR7A | MR7A36 | 56 | 57 | 1 | 434.28 | 434.28 | 27C |
| MR7A | MR7A6 | 56 | 58 | 1 | 338.06 | 338.06 | 27C |
| MR7A | MR7A1 | 56 | 59 | 1.1 | 842.46 | 765.87 | 27C |
| MR7A | MR7A22 | 56 | 60 | 1.4 | 346.31 | 247.36 | 27C |
| MR7A | MR7A23 | 56 | 61 | 1.5 | 422.10 | 281.40 | 27C |
| MR7A | MR7A255 | 56 | 62 | 1 | 296.87 | 296.87 | 27C |
| MR7A | MR7A14 | 56 | 63 | 1.4 | 251.03 | 179.30 | 27C |
| MR7A | MR7A34 | 56 | 64 | 1.4 | 199.79 | 142.70 | 27C |
| MR7A | MR7A67 | 56 | 65 | 2.3 | 255.74 | 111.19 | 27C |
| MR7A | MR7A26 | 56 | 66 | 2.6 | 322.30 | 123.96 | 27C |
| MR7A | MR7A24 | 56 | 67 | 2.2 | 423.08 | 192.31 | 27C |
| MR7A | MR7A30 | 56 | 68 | 2.5 | 772.94 | 309.18 | 27C |
| MR7A | MR7A44 | 56 | 69 | 2.1 | 570.84 | 271.83 | 27C |
| MR7A | MR7A55 | 56 | 70 | 2.5 | 888.95 | 355.58 | 27C |
| MR7A | MR7A65 | 56 | 71 | 3 | 459.21 | 153.07 | 27C |
| MR7A | MR7A251 | 56 | 72 | 2.8 | 731.19 | 261.14 | 27C |
| MR7A | MR7A260 | 56 | 73 | 3.6 | 983.02 | 273.06 | 27C |
| MR7A | MR7A264 | 56 | 74 | 3.3 | 619.71 | 187.79 | 27C |
| MR7A | MR7A270 | 56 | 75 | 2.7 | 952.65 | 352.83 | 27C |
| OLI9A | OLI9A17 | 52 | 55 | 1.6 | 314.76 | 196.73 | 27C |
| OLI9A | OLI9A8 | 52 | 56 | 1.5 | 318.50 | 212.33 | 27C |
| OLI9A | OLI9A12 | 52 | 57 | 1.3 | 540.52 | 415.78 | 27C |
| OLI9A | OLI9A3 | 52 | 58 | 2.2 | 751.76 | 341.71 | 27C |
| OLI9A | OLI9A23 | 52 | 59 | 2 | 423.40 | 211.70 | 27C |
| OLI9A | OLI9A9 | 52 | 60 | 2.6 | 794.77 | 305.68 | 27C |
| OLI9A | OLI9A30 | 52 | 61 | 2.4 | 649.40 | 270.59 | 27C |
| OLI9A | OLI9A33 | 52 | 62 | 2.1 | 658.81 | 313.72 | 27C |
| OLI9A | OLI9A38 | 52 | 63 | 2.1 | 818.26 | 389.65 | 27C |
| OLI9A | OLI9A44 | 52 | 64 | 1 | 490.34 | 490.34 | 27C |
| OLI9A | OLI9A58 | 52 | 65 | 3.5 | 281.50 | 80.43 | 27C |
| OLI9A | OLI9A18 | 52 | 66 | 2.9 | 1446.15 | 498.67 | 27C |
| OLI9A | OLI9A5 | 52 | 67 | 2.1 | 1098.72 | 523.20 | 27C |
| OLI9A | OLI9A28 | 52 | 68 | 3.1 | 464.20 | 149.74 | 27C |
| OLI9A | OLI9A32 | 52 | 69 | 3.1 | 346.31 | 111.71 | 27C |
| OLI9A | OLI9A36 | 52 | 70 | 3 | 368.12 | 122.71 | 27C |
| OLI9A | OLI9A51 | 52 | 71 | 1.7 | 649.40 | 382.00 | 27C |
| OLI9A | OLI9A60 | 52 | 72 | 3.7 | 404.82 | 109.41 | 27C |
| OLI9A | OLI9A68 | 52 | 73 | 3.5 | 339.38 | 96.97 | 27C |
| SBF9A | SBF9A1 | 56 | 55 | 1 | 341.15 | 341.15 | 27C |
| SBF9A | SBF9A8 | 56 | 56 | 1.4 | 676.23 | 483.02 | 27C |
| SBF9A | SBF9A18 | 56 | 57 | 1.7 | 893.21 | 525.42 | 27C |
| SBF9A | SBF9A17 | 56 | 58 | 1.8 | 587.09 | 326.16 | 27C |
| SBF9A | SBF9A32 | 56 | 59 | 1.6 | 636.96 | 398.10 | 27C |
| SBF9A | SBF9A31 | 56 | 60 | 1.6 | 893.21 | 558.26 | 27C |
| SBF9A | SBF9A40 | 56 | 61 | 1.6 | 214.19 | 133.87 | 27C |
| SBF9A | SBF9A3 | 56 | 62 | 2.2 | 2403.78 | 1092.63 | 27C |
| SBF9A | SBF9A10 | 56 | 63 | 1.7 | 223.82 | 131.66 | 27C |
| SBF9A | SBF9A15 | 56 | 64 | 1.5 | 284.82 | 189.88 | 27C |
| SBF9A | SBF9A25 | 56 | 65 | 2 | 446.94 | 223.47 | 27C |
| SBF9A | SBF9A42 | 56 | 66 | 3.5 | 278.65 | 79.62 | 27C |
| SBF9A | SBF9A50 | 56 | 67 | 3 | 387.30 | 129.10 | 27C |
| SBF9A | SBF9A22 | 56 | 68 | 1.6 | 897.07 | 560.67 | 27C |
| SBF9A | SBF9A29 | 56 | 69 | 2.2 | 867.62 | 394.37 | 27C |
| FLW1A | FLW1A71 | 57 | 55 | 1.6 | 314.76 | 196.73 | 30C |
| FLW1A | FLW1A75 | 57 | 56 | 1.8 | 363.62 | 202.01 | 30C |
| FLW1A | FLW1A165 | 57 | 57 | 0.9 | 186.02 | 206.69 | 30C |
| FLW1A | FLW1A166 | 57 | 58 | 0.9 | 657.39 | 730.43 | 30C |
| FLW1A | FLW1A174 | 57 | 59 | 1.6 | 346.31 | 216.44 | 30C |
| FLW1A | FLW1A179 | 57 | 60 | 1.3 | 83.74 | 64.42 | 30C |
| FLW1A | FLW1A196 | 57 | 61 | 2.2 | 1316.63 | 598.47 | 30C |
| FLW1A | FLW1A194 | 57 | 62 | 2.2 | 304.15 | 138.25 | 30C |
| FLW1A | FLW1A74 | 57 | 63 | 2.8 | 183.49 | 65.53 | 30C |
| FLW1A | FLW1A78 | 57 | 64 | 2 | 707.92 | 353.96 | 30C |
| FLW1A | FLW1A201 | 57 | 65 | 2.7 | 658.81 | 244.00 | 30C |
| FLW1A | FLW1A69 | 57 | 66 | 2.7 | 350.53 | 129.83 | 30C |
| FLW1A | FLW1A232 | 57 | 67 | 1.4 | 200.48 | 143.20 | 30C |
| FLW1A | FLW1A233 | 57 | 68 | 2.2 | 368.12 | 167.33 | 30C |
| FLW1A | FLW1A80 | 57 | 69 | 1.4 | 540.52 | 386.09 | 30C |
| FLW1A | FLW1A181 | 57 | 70 | 2.9 | 266.72 | 91.97 | 30C |
| FLW1A | FLW1A198 | 57 | 71 | 2.4 | 244.35 | 101.81 | 30C |
| FLW1A | FLW1A197 | 57 | 72 | 2.4 | 566.89 | 236.20 | 30C |
| FLW6A | FLW6A153 | 55 | 55 | 1.4 | 164.13 | 117.23 | 30C |
| FLW6A | FLW6A168 | 55 | 56 | 1.1 | 310.88 | 282.62 | 30C |
| FLW6A | FLW6A183 | 55 | 57 | 0.9 | 255.74 | 284.16 | 30C |
| FLW6A | FLW6A202 | 55 | 58 | 1.6 | 223.82 | 139.89 | 30C |
| FLW6A | FLW6A213 | 55 | 59 | 1.2 | 239.05 | 199.21 | 30C |
| FLW6A | FLW6A209 | 55 | 60 | 1.3 | 297.56 | 228.89 | 30C |
| FLW6A | FLW6A157 | 55 | 61 | 2.3 | 214.19 | 93.13 | 30C |
| FLW6A | FLW6A178 | 55 | 62 | 1.5 | 297.56 | 198.37 | 30C |
| FLW6A | FLW6A179 | 55 | 63 | 1.4 | 272.62 | 194.73 | 30C |
| FLW6A | FLW6A193 | 55 | 65 | 2 | 488.85 | 244.43 | 30C |
| FLW6A | FLW6A201 | 55 | 66 | 1.6 | 654.72 | 409.20 | 30C |
| FLW6A | FLW6A225 | 55 | 68 | 2.9 | 947.28 | 326.65 | 30C |
| FLW6A | FLW6A221 | 55 | 69 | 1.5 | 160.49 | 106.99 | 30C |
| FLW6A | FLW6A190 | 55 | 70 | 1.9 | 339.38 | 178.62 | 30C |
| FLW6A | FLW6A166 | 55 | 71 | 2.9 | 1066.43 | 367.73 | 30C |
| FLW6A | FLW6A171 | 55 | 72 | 3.2 | 204.96 | 64.05 | 30C |
| FLW6A | FLW6A185 | 55 | 73 | 2.6 | 540.52 | 207.89 | 30C |
| MR10A | MR10A83 | 60 | 55 | 1.2 | 315.81 | 263.18 | 30C |
| MR10A | MR10A86 | 60 | 56 | 1.6 | 359.18 | 224.49 | 30C |
| MR10A | MR10106 | 60 | 57 | 1.6 | 273.54 | 170.96 | 30C |
| MR10A | MR10A60 | 60 | 58 | 1 | 217.62 | 217.62 | 30C |
| MR10A | MR10A111 | 60 | 59 | 2.2 | 474.65 | 215.75 | 30C |
| MR10A | MR10A96 | 60 | 60 | 1.2 | 307.45 | 256.21 | 30C |
| MR10A | MR10A113 | 60 | 61 | 1.2 | 326.15 | 271.79 | 30C |
| MR10A | MR10A56 | 60 | 62 | 1.5 | 303.87 | 202.58 | 30C |
| MR10A | MR10A87 | 60 | 63 | 0.9 | 233.89 | 259.88 | 30C |
| MR10A | MR10A94 | 60 | 64 | 1.4 | 525.99 | 375.71 | 30C |
| MR10A | MR10A188 | 60 | 65 | 1.7 | 498.15 | 293.03 | 30C |
| MR10A | MR10A190 | 60 | 66 | 1.2 | 731.19 | 609.33 | 30C |
| MR10A | MR10A192 | 60 | 67 | 1.6 | 334.03 | 208.77 | 30C |
| MR10A | MR10A99 | 60 | 68 | 2.6 | 342.15 | 131.60 | 30C |
| MR10A | MR10A100 | 60 | 69 | 2.6 | 459.21 | 176.62 | 30C |
| MR10A | MR10A197 | 60 | 70 | 3 | 193.46 | 64.49 | 30C |
| MR10A | MR10A202 | 60 | 71 | 2.5 | 396.79 | 158.72 | 30C |
| MR10A | MR10A239 | 60 | 72 | 2.7 | 474.65 | 175.80 | 30C |
| MR10A | MR10A51 | 60 | 73 | 1.9 | 382.09 | 201.10 | 30C |
| MR10A | MR10A92 | 60 | 74 | 1.6 | 250.02 | 156.26 | 30C |
| MR10A | MR10A231 | 60 | 75 | 1.4 | 554.77 | 396.26 | 30C |
| MR7A | MR7A157 | 57 | 55 | 1.1 | 342.15 | 311.05 | 30C |
| MR7A | MR7A167 | 57 | 56 | 1 | 231.06 | 231.06 | 30C |
| MR7A | MR7A155 | 57 | 57 | 0.3 | 191.02 | 636.72 | 30C |
| MR7A | MR7A199 | 57 | 58 | 1.3 | 386.91 | 297.62 | 30C |
| MR7A | MR7A209 | 57 | 59 | 1 | 602.95 | 602.95 | 30C |
| MR7A | MR7A213 | 57 | 60 | 1.6 | 503.51 | 314.69 | 30C |
| MR7A | MR7A220 | 57 | 61 | 1.3 | 398.17 | 306.28 | 30C |
| MR7A | MR7A240 | 57 | 62 | 1.1 | 364.92 | 331.74 | 30C |
| MR7A | MR7A158 | 57 | 63 | 2.7 | 743.90 | 275.52 | 30C |
| MR7A | MR7A174 | 57 | 64 | 2.8 | 434.63 | 155.23 | 30C |
| MR7A | MR7A202 | 57 | 65 | 2.5 | 440.04 | 176.01 | 30C |
| MR7A | MR7A215 | 57 | 66 | 3 | 434.28 | 144.76 | 30C |
| MR7A | MR7A217 | 57 | 67 | 2.4 | 428.63 | 178.59 | 30C |
| MR7A | MR7A221 | 57 | 68 | 2.6 | 275.02 | 105.78 | 30C |
| MR7A | MR7A303 | 57 | 69 | 1.5 | 306.33 | 204.22 | 30C |
| MR7A | MR7A204 | 57 | 70 | 2.4 | 567.01 | 236.25 | 30C |
| MR7A | MR7A207 | 57 | 71 | 2.9 | 1449.90 | 499.96 | 30C |
| MR7A | MR7A228 | 57 | 72 | 3 | 428.63 | 142.88 | 30C |
| MR7A | MR7A316 | 57 | 73 | 2.9 | 1597.61 | 51.72 | 30C |
| MR7A | MR7A322 | 57 | 74 | 2 | 2843.66 | 1421.83 | 30C |
| MR7A | MR7A325 | 57 | 75 | 2.2 | 503.51 | 228.87 | 30C |
| OLI9A | OLI9A90 | 52 | 55 | 3.6 | 270.72 | 75.20 | 30C |
| OLI9A | OLI9A89 | 52 | 56 | 1.8 | 326.15 | 181.19 | 30C |
| OLI9A | OLI9A80 | 52 | 57 | 1.3 | 423.40 | 325.69 | 30C |
| OLI9A | OLI9A77 | 52 | 58 | 2.8 | 995.32 | 355.47 | 30C |
| OLI9A | OLI9A79 | 52 | 59 | 2.5 | 1024.03 | 409.61 | 30C |
| OLI9A | OLI9A86 | 52 | 60 | 2.2 | 1148.27 | 521.94 | 30C |
| OLI9A | OLI9A97 | 52 | 61 | 2.9 | 386.74 | 133.36 | 30C |
| OLI9A | OLI9A108 | 52 | 62 | 2.6 | 511.87 | 196.87 | 30C |
| OLI9A | OLI9A116 | 52 | 63 | 1.8 | 460.89 | 256.05 | 30C |
| OLI9A | OLI9A132 | 52 | 64 | 2 | 550.37 | 275.18 | 30C |
| OLI9A | OLI9A140 | 52 | 65 | 3.5 | 790.89 | 225.97 | 30C |
| OLI9A | OLI9A134 | 52 | 66 | 2.6 | 841.17 | 323.53 | 30C |
| OLI9A | OLI9A136 | 52 | 67 | 3.4 | 346.92 | 102.03 | 30C |
| OLI9A | OLI9A87 | 52 | 68 | 3 | 554.77 | 184.92 | 30C |
| OLI9A | OLI9A112 | 52 | 69 | 2.1 | 731.19 | 348.19 | 30C |
| OLI9A | OLI9A128 | 52 | 70 | 2.6 | 441.90 | 169.96 | 30C |
| OLI9A | OLI9A138 | 52 | 71 | 3 | 580.00 | 193.33 | 30C |
| OLI9A | OLI9A146 | 52 | 72 | 3.5 | 2108.30 | 602.37 | 30C |
| SBF9A | SBF9A153 | 58 | 55 | 1.4 | 282.54 | 201.81 | 30C |
| SBF9A | SBF9A163 | 58 | 56 | 1.9 | 728.65 | 383.50 | 30C |
| SBF9A | SBF9A101 | 58 | 57 | 1.6 | 239.05 | 149.41 | 30C |
| SBF9A | SBF9A128 | 58 | 59 | 2 | 249.76 | 124.88 | 30C |
| SBF9A | SBF9A144 | 58 | 60 | 1.7 | 947.28 | 557.22 | 30C |
| SBF9A | SBF9A146 | 58 | 61 | 2.2 | 395.96 | 179.98 | 30C |
| SBF9A | SBF9A132 | 58 | 62 | 1.7 | 657.39 | 386.70 | 30C |
| SBF9A | SBF9A133 | 58 | 62 | 1.9 | 266.72 | 140.38 | 30C |
| SBF9A | SBF9A140 | 58 | 63 | 1.7 | 362.52 | 213.25 | 30C |
| SBF9A | SBF9A158 | 58 | 64 | 2.2 | 483.83 | 219.92 | 30C |
| SBF9A | SBF9A166 | 58 | 65 | 2.3 | 567.01 | 246.53 | 30C |
| SBF9A | SBF9A172 | 58 | 66 | 1.9 | 457.98 | 241.04 | 30C |
| SBF9A | SBF9A175 | 58 | 67 | 2.9 | 404.82 | 139.59 | 30C |
| SBF9A | SBF9A111 | 58 | 68 | 2.9 | 749.40 | 258.41 | 30C |
| SBF9A | SBF9A137 | 58 | 69 | 2.8 | 54.86 | 19.59 | 30C |
| FLW1A | FLW1A67 | 63 | 55 | 1.2 | 319.54 | 266.28 | 33C |
| FLW1A | FLW1A102 | 63 | 56 | 1 | 772.07 | 772.07 | 33C |
| FLW1A | FLW1A110 | 63 | 57 | 1.1 | 1786.42 | 1624.02 | 33C |
| FLW1A | FLW1A119 | 63 | 58 | 0.9 | 400.56 | 445.07 | 33C |
| FLW1A | FLW1A108 | 63 | 59 | 0.9 | 346.31 | 384.79 | 33C |
| FLW1A | FLW1A132 | 63 | 60 | 1.1 | 187.61 | 170.55 | 33C |
| FLW1A | FLW1A131 | 63 | 60 | 1.1 | 157.64 | 143.31 | 33C |
| FLW1A | FLW1A134 | 63 | 62 | 1.6 | 231.06 | 144.41 | 33C |
| FLW1A | FLW1A117 | 63 | 63 | 1.7 | 387.30 | 227.83 | 33C |
| FLW1A | FLW1A139 | 63 | 64 | 1.5 | 244.35 | 162.90 | 33C |
| FLW1A | FLW1A152 | 63 | 65 | 1.2 | 983.02 | 819.19 | 33C |
| FLW1A | FLW1A239 | 63 | 66 | 1 | 555.46 | 555.46 | 33C |
| FLW1A | FLW1A97 | 63 | 67 | 0.7 | 88.71 | 126.73 | 33C |
| FLW1A | FLW1A143 | 63 | 68 | 1.6 | 1357.42 | 848.39 | 33C |
| FLW1A | FLW1A248 | 63 | 69 | 1.2 | 570.84 | 475.70 | 33C |
| FLW1A | FLW1A247 | 63 | 70 | 2 | 264.61 | 132.31 | 33C |
| FLW6A | FLW6A76 | 58 | 55 | 1.1 | 278.65 | 253.32 | 33C |
| FLW6A | FLW6A83 | 58 | 56 | 1.1 | 179.45 | 163.14 | 33C |
| FLW6A | FLW6A85 | 58 | 57 | 1.1 | 338.11 | 307.38 | 33C |
| FLW6A | FLW6A92 | 58 | 58 | 1 | 200.48 | 200.48 | 33C |
| FLW6A | FLW6A99 | 58 | 59 | 0.9 | 540.52 | 600.58 | 33C |
| FLW6A | FLW6A125 | 58 | 60 | 0.9 | 389.40 | 432.67 | 33C |
| FLW6A | FLW6A79 | 58 | 61 | 1.3 | 579.18 | 445.53 | 33C |
| FLW6A | FLW6A81 | 58 | 62 | 1.7 | 1035.18 | 608.93 | 33C |
| FLW6A | FLW6A86 | 58 | 63 | 1.3 | 534.25 | 410.96 | 33C |
| FLW6A | FLW6A96 | 58 | 64 | 0.7 | 531.75 | 759.64 | 33C |
| FLW6A | FLW6A111 | 58 | 65 | 1.2 | 511.87 | 426.56 | 33C |
| FLW6A | FLW6A109 | 58 | 66 | 0.5 | 202.77 | 405.55 | 33C |
| FLW6A | FLW6A133 | 58 | 67 | 1 | 50.78 | 50.78 | 33C |
| FLW6A | FLW6A143 | 58 | 68 | 1.4 | 509.51 | 363.94 | 33C |
| FLW6A | FLW6A142 | 58 | 69 | 1 | 156.92 | 156.92 | 33C |
| MR10A | MR10A119 | 61 | 55 | 0.5 | 216.04 | 432.07 | 33C |
| MR10A | MR10A123 | 61 | 56 | 1.1 | 474.65 | 431.50 | 33C |
| MR10A | MR10A125 | 61 | 57 | 1.1 | 237.08 | 215.52 | 33C |
| MR10A | MR10A128 | 61 | 58 | 1.4 | 173.27 | 123.76 | 33C |
| MR10A | MR10A137 | 61 | 59 | 1.1 | 130.30 | 118.46 | 33C |
| MR10A | MR10A150 | 61 | 60 | 1.2 | 82.26 | 68.55 | 33C |
| MR10A | MR10A154 | 61 | 61 | 1.1 | 231.06 | 210.06 | 33C |
| MR10A | MR10A126 | 61 | 62 | 1 | 208.91 | 208.91 | 33C |
| MR10A | MR10A162 | 61 | 63 | 1.5 | 940.50 | 627.00 | 33C |
| MR10A | MR10A175 | 61 | 64 | 1.6 | 817.26 | 510.79 | 33C |
| MR10A | MR10A216 | 61 | 65 | 1.5 | 840.44 | 560.29 | 33C |
| MR10A | MR10A134 | 61 | 66 | 1.4 | 350.51 | 250.37 | 33C |
| MR10A | MR10A138 | 61 | 67 | 1.5 | 208.67 | 139.11 | 33C |
| MR10A | MR10A151 | 61 | 68 | 2.3 | 251.03 | 109.14 | 33C |
| MR10A | MR10A160 | 61 | 69 | 1.6 | 270.72 | 169.20 | 33C |
| MR10A | MR10A157 | 61 | 70 | 2.2 | 187.61 | 85.28 | 33C |
| MR10A | MR10A167 | 61 | 71 | 1.9 | 211.50 | 111.32 | 33C |
| MR10A | MR10A224 | 61 | 72 | 1.9 | 422.10 | 222.16 | 33C |
| MR10A | MR10A164 | 61 | 73 | 0.8 | 658.81 | 823.51 | 33C |
| MR10A | MR10A214 | 61 | 74 | 1 | 386.74 | 386.74 | 33C |
| MR10A | MR10A220 | 61 | 75 | 1.1 | 1740.08 | 1581.89 | 33C |
| MR7A | MR7A101 | 61 | 55 | 0.8 | 191.02 | 238.77 | 33C |
| MR7A | MR7A116 | 61 | 56 | 0.5 | 74.25 | 148.50 | 33C |
| MR7A | MR7A104 | 61 | 57 | 0.5 | 330.06 | 660.11 | 33C |
| MR7A | MR7A113 | 61 | 58 | 0.5 | 252.86 | 505.72 | 33C |
| MR7A | MR7A128 | 61 | 59 | 0.9 | 233.89 | 259.88 | 33C |
| MR7A | MR7A138 | 61 | 60 | 0.7 | 281.50 | 402.15 | 33C |
| MR7A | MR7A131 | 61 | 61 | 0.6 | 205.56 | 342.60 | 33C |
| MR7A | MR7A192 | 61 | 62 | 0.8 | 103.63 | 129.54 | 33C |
| MR7A | MR7A103 | 61 | 63 | 1.2 | 237.08 | 197.56 | 33C |
| MR7A | MR7A120 | 61 | 64 | 1.6 | 265.81 | 166.13 | 33C |
| MR7A | MR7A114 | 61 | 65 | 1.9 | 550.37 | 289.67 | 33C |
| MR7A | MR7A124 | 61 | 66 | 1.2 | 359.18 | 299.32 | 33C |
| MR7A | MR7A121 | 61 | 67 | 2.2 | 255.74 | 116.25 | 33C |
| MR7A | MR7A187 | 61 | 68 | 1.9 | 341.19 | 179.57 | 33C |
| MR7A | MR7A82 | 61 | 69 | 1.5 | 139.21 | 92.81 | 33C |
| MR7A | MR7A84 | 61 | 70 | 1.5 | 751.76 | 501.17 | 33C |
| MR7A | MR7A180 | 61 | 71 | 1.4 | 390.44 | 278.89 | 33C |
| MR7A | MR7A289 | 61 | 72 | 1.9 | 282.54 | 148.71 | 33C |
| MR7A | MR7A300 | 61 | 73 | 2.6 | 867.62 | 333.70 | 33C |
| MR7A | MR7A293 | 61 | 74 | 1.8 | 518.72 | 288.18 | 33C |
| MR7A | MR7A178 | 61 | 75 | 1.6 | 586.66 | 366.67 | 33C |
| OLI9A | OLI9A160 | 53 | 55 | 1.6 | 338.06 | 211.29 | 33C |
| OLI9A | OLI9A150 | 53 | 56 | 1.2 | 176.57 | 147.14 | 33C |
| OLI9A | OLI9A163 | 53 | 57 | 1.7 | 864.33 | 508.43 | 33C |
| OLI9A | OLI9A151 | 53 | 58 | 1.9 | 332.12 | 174.80 | 33C |
| OLI9A | OLI9A152 | 53 | 59 | 1.6 | 332.12 | 207.58 | 33C |
| OLI9A | OLI9A188 | 53 | 60 | 1.8 | 654.72 | 363.73 | 33C |
| OLI9A | OLI9A191 | 53 | 61 | 1.9 | 359.18 | 189.04 | 33C |
| OLI9A | OLI9A195 | 53 | 62 | 2.1 | 534.25 | 254.40 | 33C |
| OLI9A | OLI9A199 | 53 | 63 | 1.8 | 391.81 | 217.67 | 33C |
| OLI9A | OLI9A210 | 53 | 64 | 1.9 | 233.67 | 122.98 | 33C |
| OLI9A | OLI9A170 | 53 | 65 | 1.6 | 447.56 | 279.72 | 33C |
| OLI9A | OLI9A203 | 53 | 66 | 1.9 | 699.94 | 368.39 | 33C |
| OLI9A | OLI9A194 | 53 | 67 | 1.2 | 867.44 | 722.86 | 33C |
| OLI9A | OLI9A216 | 53 | 68 | 1.1 | 772.07 | 701.88 | 33C |
| OLI9A | OLI9A184 | 53 | 69 | 1.6 | 160.49 | 100.30 | 33C |
| OLI9A | OLI9A166 | 53 | 70 | 2.6 | 602.95 | 231.90 | 33C |
| OLI9A | OLI9A169 | 53 | 71 | 1.8 | 1583.86 | 879.92 | 33C |
| OLI9A | OLI9A193 | 53 | 72 | 2.1 | 474.67 | 226.03 | 33C |
| OLI9A | OLI9A206 | 53 | 73 | 1.7 | 319.54 | 187.96 | 33C |
| SBF9A | SBF9A52 | 61 | 55 | 1.1 | 550.37 | 500.33 | 33C |
| SBF9A | SBF9A61 | 61 | 56 | 1.1 | 357.76 | 325.24 | 33C |
| SBF9A | SBF9A58 | 61 | 57 | 1.4 | 562.90 | 402.07 | 33C |
| SBF9A | SBF9A67 | 61 | 58 | 1.5 | 842.46 | 561.64 | 33C |
| SBF9A | SBF9A86 | 61 | 59 | 0.9 | 103.61 | 115.13 | 33C |
| SBF9A | SBF9A94 | 61 | 60 | 1 | 191.81 | 191.81 | 33C |
| SBF9A | SBF9A99 | 61 | 61 | 1.5 | 947.28 | 631.52 | 33C |
| SBF9A | SBF9A56 | 61 | 62 | 1 | 405.55 | 405.55 | 33C |
| SBF9A | SBF9A57 | 61 | 63 | 0.8 | 324.81 | 406.02 | 33C |
| SBF9A | SBF9A88 | 61 | 64 | 1.2 | 332.02 | 276.68 | 33C |
| SBF9A | SBF9A106 | 61 | 65 | 1.5 | 1443.32 | 962.21 | 33C |
| SBF9A | SBF9A104 | 61 | 66 | 1.2 | 518.63 | 432.19 | 33C |
| SBF9A | SBF9A113 | 61 | 67 | 1.5 | 423.18 | 282.12 | 33C |
| SBF9A | SBF9A118 | 61 | 68 | 1.5 | 1399.63 | 933.09 | 33C |
| SBF9A | SBF9A123 | 61 | 69 | 2 | 579.18 | 289.59 | 33C |
