# Supplementary material for: Ecdysteroid responses to urban heat island conditions during development of the western black widow spider (Latrodectus hesperus)
Source: PLoS One. 2022 Apr 28;17(4):e0267398. doi: 10.1371/journal.pone.0267398 (PMC9049550; doi:10.1371/journal.pone.0267398)
Supplement: Attachment — Submitted filename: List of Responses.docx [file pone.0267398.s004.docx]

Response to Reviewers – List of Responses

**Journal Requirements:**

When submitting your revision, we need you to address these additional requirements.

1. Please ensure that your manuscript meets PLOS ONE's style requirements, including those for file naming. The PLOS ONE style templates can be found at

<https://journals.plos.org/plosone/s/file?id=wjVg/PLOSOne_formatting_sample_main_body.pdf> and

<https://journals.plos.org/plosone/s/file?id=ba62/PLOSOne_formatting_sample_title_authors_affiliations.pdf>

The manuscript has been reformatted to meet journal style requirements.

2. Thank you for stating the following financial disclosure:

“This work was supported by an ASU New College Undergraduate Interdisciplinary Research Experience (NCUIRE; https://newcollege.asu.edu/ncuire) award to CM, a Salt River Project Life Sciences Scholarship (https://scholarships.asu.edu/scholarship/94) to CM, a Central Arizona-Phoenix Long Term Ecological Research (CAP-LTER; https://sustainability-innovation.asu.edu/caplter/) award to CJ, and an ASU New College Scholarship, Research, and Creative Activities award to JHP and CJ.”

Please state what role the funders took in the study. If the funders had no role, please state: "The funders had no role in study design, data collection and analysis, decision to publish, or preparation of the manuscript."

If this statement is not correct you must amend it as needed.

Please include this amended Role of Funder statement in your cover letter; we will change the online submission form on your behalf.

The funders had no role in study design, data collection and analysis, decision to publish, or preparation of the manuscript.

3. In your Data Availability statement, you have not specified where the minimal data set underlying the results described in your manuscript can be found. PLOS defines a study's minimal data set as the underlying data used to reach the conclusions drawn in the manuscript and any additional data required to replicate the reported study findings in their entirety. All PLOS journals require that the minimal data set be made fully available. For more information about our data policy, please see http://journals.plos.org/plosone/s/data-availability.

Upon re-submitting your revised manuscript, please upload your study’s minimal underlying data set as either Supporting Information files or to a stable, public repository and include the relevant URLs, DOIs, or accession numbers within your revised cover letter. For a list of acceptable repositories, please see http://journals.plos.org/plosone/s/data-availability#loc-recommended-repositories. Any potentially identifying patient information must be fully anonymized.

Important: If there are ethical or legal restrictions to sharing your data publicly, please explain these restrictions in detail. Please see our guidelines for more information on what we consider unacceptable restrictions to publicly sharing data: http://journals.plos.org/plosone/s/data-availability#loc-unacceptable-data-access-restrictions. Note that it is not acceptable for the authors to be the sole named individuals responsible for ensuring data access.

We will update your Data Availability statement to reflect the information you provide in your cover letter.

The minimal data set has been included as a Supporting Information File:

S1 Table. Spider ecdysteroid and developmental measurements

4. Please review your reference list to ensure that it is complete and correct. If you have cited papers that have been retracted, please include the rationale for doing so in the manuscript text, or remove these references and replace them with relevant current references. Any changes to the reference list should be mentioned in the rebuttal letter that accompanies your revised manuscript. If you need to cite a retracted article, indicate the article’s retracted status in the References list and also include a citation and full reference for the retraction notice.

The reference list in our manuscript is complete and accurate and has been cross-checked against the Retraction Watch Database. The reference list does not include any retracted articles.

**Additional Editor Comments:**

**Reviewer 1**

The authors explored the responses to temperature during development of the Western Black Widow Spider. The research methodologies are reasonable, and the findings are interesting. However, there are still a few aspects that should be improved to make the paper publishable. The paper is not well-organized. I focus here only on some points, which are hopefully easy for the authors to take into account in the revision.

1. Introduction- The author introduced the negative impacts of UHI. However, relevant research on UHI is lacking. Some important relevant references should be cited as follows.

Spatial Variability and Temporal Heterogeneity of Surface Urban Heat Island Patterns and the Suitability of Local Climate Zones for Land Surface Temperature Characterization. DOI: 10.3390/rs13214338.

Influence of urban morphological characteristics on thermal environment, Sustainable Cities and Society (2021), https://doi.org/10.1016/j.scs.2021.103045.

Contribution of urban ventilation to the thermal environment and urban energy demand: Different climate background perspectives, Science of the Total Environment (2021), https://doi.org/10.1016/j.scitotenv.2021.148791.

Suitability of human settlements in mountainous areas from the perspective of ventilation: a case study of the main urban area of Chongqing, Journal of Cleaner Production (2021), https://doi.org/10.1016/j.jclepro.2021.127467.

The impact of urban renewal on land surface temperature changes: A case study in the main city of Guangzhou, China. Remote Sensing (2020), https://doi.org/10.3390/rs12050794.

Impacts of Neighboring Buildings on the Cold Island Effect of Central Parks: A Case Study of Beijing, China. Sustainability (2020), doi: 10.3390/su12229499.

Additional detail regarding the influence of urban morphology on urban temperatures has been included and the references listed have now been included.

2. The author emphasizes UHI, while temperature was chosen in this manuscript. 27 ˚C, 33˚C and 37 ˚C can represent UHI? It is more appropriate to explore the effects of temperature.

The rational for using specific temperatures has been included with appropriate citation (introduction paragraph 3). Additional detail has been added to the methods section (methods; page 7).

3. Figure 2 is not clear enough, please modify it.

Figure 2 has been modified for clarity, presenting the results for each temperature as separate graphs and indicating the location of the pre-molt peak that occurs 2 days prior to molt 2.

4. There is no Conclusion in this manuscript, please confirm it.

The last paragraph of the discussion has been reformatted to more concisely summarize key findings as a Conclusion Section.

**Reviewer 2**

After a very careful reading of the work entitled "Ecdysteroid Responses to Urban Heat Island Conditions during Development of the Western Black Widow Spider", I have found a very well-done work, well presented, and organized, clear in concepts and methodology. The topic and context attract attention for many readers from various disciplines. The study is worth to be published after conducting the revisions.

I just suggest the author(s) add uncertainty analysis to enhance the scientific rigor of the research.

This paper addresses the question of how ecdysteroid measurements recorded from spiderlings reared at different temperatures compare to one another rather than addressing the absolute uncertainties of ecdysteroid measurement techniques. We therefore give results with statistical uncertainties (mean, standard error), rather than completing additional uncertainty analysis, which we believe is outside the scope of this paper.

**Additional Comments:**

While revising your submission, please upload your figure files to the Preflight Analysis and Conversion Engine (PACE) digital diagnostic tool, https://pacev2.apexcovantage.com/. PACE helps ensure that figures meet PLOS requirements. To use PACE, you must first register as a user. Registration is free. Then, login and navigate to the UPLOAD tab, where you will find detailed instructions on how to use the tool. If you encounter any issues or have any questions when using PACE, please email PLOS at figures@plos.org. Please note that Supporting Information files do not need this step.

All figures were converted using PACE to ensure compliance with PLOS requirements.
